# Radiological Diagnosis of Chronic Liver Disease and Hepatocellular Carcinoma: A Review

**DOI:** 10.1007/s10916-023-01968-7

**Published:** 2023-07-11

**Authors:** Sonit Singh, Shakira Hoque, Amany Zekry, Arcot Sowmya

**Affiliations:** 1 School of CSE, UNSW Sydney, High St, Kensington, 2052 NSW Australia; 2 St George and Sutherland Clinical Campus, School of Clinical Medicine, UNSW, High St, Kensington, 2052 NSW Australia; 3 Gastroenterology and Hepatology Department, St George Hospital, Hogben St, Kogarah, 2217 NSW Australia

**Keywords:** Liver diseases, Ultrasound imaging, Medical imaging, Machine learning, Deep learning

## Abstract

Medical image analysis plays a pivotal role in the evaluation of diseases, including screening, surveillance, diagnosis, and prognosis. Liver is one of the major organs responsible for key functions of metabolism, protein and hormone synthesis, detoxification, and waste excretion. Patients with advanced liver disease and Hepatocellular Carcinoma (HCC) are often asymptomatic in the early stages; however delays in diagnosis and treatment can lead to increased rates of decompensated liver diseases, late-stage HCC, morbidity and mortality. Ultrasound (US) is commonly used imaging modality for diagnosis of chronic liver diseases that includes fibrosis, cirrhosis and portal hypertension. In this paper, we first provide an overview of various diagnostic methods for stages of liver diseases and discuss the role of Computer-Aided Diagnosis (CAD) systems in diagnosing liver diseases. Second, we review the utility of machine learning and deep learning approaches as diagnostic tools. Finally, we present the limitations of existing studies and outline future directions to further improve diagnostic accuracy, as well as reduce cost and subjectivity, while also improving workflow for the clinicians.

## Introduction

The delivery of quality healthcare is one of the primary agendas of every nation. Medical imaging includes techniques and processes designed to visualise body parts, tissue or organs for medical purposes, including both diagnostic and therapeutic. With recent advances in Artificial Intelligence (AI) and medical imaging technologies, biomedical image analysis has transformed clinical practice by providing improved insights into human anatomy and disease processes.

Liver disease progression can be characterised by histopathological and haemodynamic changes within the hepatic parenchyma, which correlates to signs found on imaging modalities. Liver fibrosis is the most common outcome of chronic liver injury. Persistent hepatic parenchymal damage results in activation of immune cells and synthesis of fibrotic extracellular matrix components leading to scar formation, which impairs cell function [1, 2]. Progressive liver fibrosis can lead to liver cirrhosis and related complications such as portal hypertension [3]. Portal hypertension in turn leads to multiple complications including splenomegaly, ascites, varices, hepatorenal syndrome and hepatic encephalopathy. Further, the process of chronic liver injury eventually leads to hepatocellular carcinoma (HCC), with cirrhosis being the main precursor of HCC [4]. Overall, one-third of cirrhotic patients will develop HCC during their lifetime. Risk factors for chronic liver disease and eventually liver cirrhosis include chronic infection with HBV or HCV, heavy alcohol intake, and metabolic liver disease [5].

According to World Health Organisation (WHO), HCC is the fourth-leading cause of cancer-related deaths in the world [6]. The prognosis of patients with this tumour remains poor, with a 5-year survival rate of 19% at time of diagnosis [7]. Unfortunately, this is because HCC is often diagnosed at its advanced stages due to the absence of symptoms in patients with early disease, and the poor adherence to surveillance in high-risk patients. The five-year survival rate for patients whose tumours are detected at an early stage and who receive treatment exceeds 70% [8]. Therefore, early diagnosis and staging of liver diseases plays a pivotal role in reducing HCC-related deaths, as well as reducing healthcare costs.

Many computational methods have been developed for the radiological diagnosis of chronic liver disease and HCC. Among the various options, Machine Learning (ML) and Deep Learning (DL) methods have received significant attention due to their outstanding performance on disease diagnosis and prognosis. In this review, we aim to perform a comprehensive analysis of various ML and DL methods for the diagnosis of chronic liver disease and HCC. We first provide an overview of methods in the ML pipeline including pre-processing, feature extraction, and learning algorithms. We then provide an overview of Convolutional Neural Networks (CNNs), which are specialised deep learning algorithms for processing 2D or 3D data. We discuss in detail the application of various methods for liver diseases such as fibrosis, cirrhosis, and HCC. We further outline limitations in current studies and provide research directions that need attention from the scientific community.

## Radiological diagnosis of fibrosis and cirrhosis

Ultrasound (US) is typically the first-line radiological study obtained in patients suspected of having cirrhosis because it is readily available, non-invasive, well-tolerated, less expensive than its CT or MRI counterparts, provides real-time image acquisition and display, and does not expose patients to the adverse effects of intravenous contrast or radiation. Changes in tissue composition in a cirrhotic liver can be detected on gray-scale US. The ultrasonographic hallmarks of cirrhosis are a nodular or irregular surface, coarsened liver edge and increased echogenicity; in advanced disease, the gross liver appears atrophied and multi-nodular (with typically atrophy of right lobe and hypertrophy of caudate or left lobes) [9]. A prospective study of 100 patients with suspected cirrhosis who underwent liver biopsy showed that high-resolution US had 91% sensitivity and 94% specificity in detecting cirrhosis [10]. In another similar study, hepatic surface nodularity, especially detected by a linear probe, was shown to be the most direct sign of advanced fibrosis, with reported sensitivity and specificity of 54% and 95% respectively [11, 12]. However, the disadvantages of US in diagnosis of cirrhosis includes high operator dependency and effect on resolution due to presence of speckle noise and fat in obese patients [13].

Ultrasonography also detects portal hypertension, which is a predictive marker of poor outcomes in cirrhosis, with reverse portal flow in decompensated cirrhosis being a poor prognostic marker [14]. Recent studies have shown that HCC incidence increases in parallel to portal pressure. B-mode US signs suggestive of increased portal pressures include increased portal vein diameter, splenomegaly, ascites and presence of abnormal collateral route; US is able to detect the onset of these complications early. Doppler US has high specificity and moderate sensitivity for the diagnosis of clinically significant portal hypertension, but is limited in detecting slow blood flow and has reduced frame rate [15, 16].

Ultrasound-based elastography is a radiological technique that is used as an alternative to liver biopsy to stage the degree of liver fibrosis. Shear wave elastography and strain elastography are the two main techniques used to evaluate liver stiffness, by essentially measuring the hepatic tissue response after mechanical excitation [17]. The accuracy of elastography has primarily been investigated in patients with chronic HCV and HBV. Overall, it has an estimated sensitivity of 70% and specificity of 85% in diagnosing significant fibrosis (F greater than or equal to 2), and 87% and 91% respectively in cirrhosis (F4). A meta-analysis of 17 studies consisting of 7,058 patients has also shown that it can be used to predict complications in chronic liver disease patients, with baseline liver stiffness associated with risk of hepatic decompensation (relative risk [RR] 1.07, 95% CI 1.03$-$1.11), HCC development (RR 1.11, 95% CI 1.05$-$1.18), and death (RR 1.22, 95% CI 1.05$-$1.43) [18]. However, it can be limited in its use in some cases where other factors affect measured liver stiffness, including elevated central venous pressures in patients with severe cardio-respiratory disease, obesity and anatomic distortion.

Given the high sensitivity of ultrasound diagnosis of cirrhosis, CT or MRI is not typically required for diagnosis. However, [12] did show that early stages of liver parenchymal abnormalities and morphological changes in the liver on MRI and CT were predictive of cirrhosis by multivariate analysis (the diagnostic accuracy being 66.0%, 71.9% and 67.9% for US, CT and MRI respectively). Furthermore, physiological parameters have been identified from measurements on multiphase CT as markers of fibrosis - for example, changes in liver perfusion, arterial fraction and mean transit time of contrast do correlate well with severity of cirrhosis (by Child-Pugh classification) [19, 20]. These techniques are yet to be validated in multi-centre trials and remain investigated at this stage.

Diffusion-weighted and contrast-enhanced MRI are able to quantify fibrosis as MRI can detect restricted movement of water that occurs in the expansion of extracellular fluid space in liver fibrosis. [21] showed this had a sensitivity of 85% and specificity of 100% for diagnosis of cirrhosis; as well as sensitivity of 89% and specificity of 80% to stage the degree of cirrhosis [22]. However, despite its strengths, the use of CT and MRI are limited in the clinical setting because they subject patients to ionizing radiation and intravenous contrast material, can significantly increase the cost of the procedure, and MRI-based techniques in particular are subject to limited availability and degree of technical expertise [23].

## Radiological diagnosis of HCC

Focal liver lesions seen on ultrasonography in both cirrhotic and non-cirrhotic patients are concerning for HCC. As per the European Association for the Study of the Liver (EASL) Clinical Practice Guidelines, HCC surveillance in at-risk patients consists of six-monthly abdominal ultrasounds. This time interval is based on the expected tumour growth rate supported by observational data, and therefore the interval is not shortened for people at higher risk of HCC. This surveillance has shown a reduction in disease-related mortality with a meta-analysis including 19 studies showing that ultrasound surveillance detected the majority of HCC tumours before they presented clinically, with a pooled sensitivity of 94% [24].

The detection of nodules in cirrhotic patients always warrants further diagnostic contrast-enhanced imaging because benign and malignant nodules are not able to be differentiated based on ultrasonographic appearance alone, with current guidelines recommending CT or MRI to further characterise lesions 1 cm or greater identified on surveillance US [25]. Also, US has a low sensitivity of 63% for detecting early-stage HCC, particularly in the instance of very coarse liver echotexture in advanced fibrosis; and therefore, the performance of US in identification of small nodules in these cases is highly dependent on operator expertise, patient factors (for example, obesity) and the quality of equipment. Reported specificity for detecting HCC with US at any stage is uniformly high at > 90% [24–28]. Ultrasound-based elastography has also been studied for the evaluation of focal liver lesions, but because of its limitations with restricted depth of penetration and inability to differentiate between stiffness of benign and malignant tissue, it is not recommended for this use.

Contrast-enhanced radiological diagnosis of HCC is based on vascular phases (that is, lesion appearance in the late arterial phase, portal venous phase, and the delayed phase). The typical hallmark of HCC is the combination of hypervascularity in the late arterial phase and washout on portal venous and/or delayed phases, which reflects the vascular derangement occurring during hepatocarcinogenesis [29]. Both CT and MRI are more sensitive than ultrasound for detecting HCC < 2 cm and thus more likely to identify candidates for liver transplantation therapy [30]. As expected, in most studies, MRI has higher sensitivity compared to CT in HCC diagnosis which does vary according to HCC size, with MRI performing better on smaller lesions (sensitivity of 48% and 62% for CT and MRI, respectively, in tumours smaller than 20 mm vs. 92% and 95% for CT and MRI, respectively, in tumours equal or larger than 20 mm) [31, 32]. CT or MRI can be considered when patient factors such as obesity, severe parenchymal heterogeneity from advanced cirrhosis, intestinal gas and chest wall deformity prevent adequate US assessment [24].

**Fig. 1:**
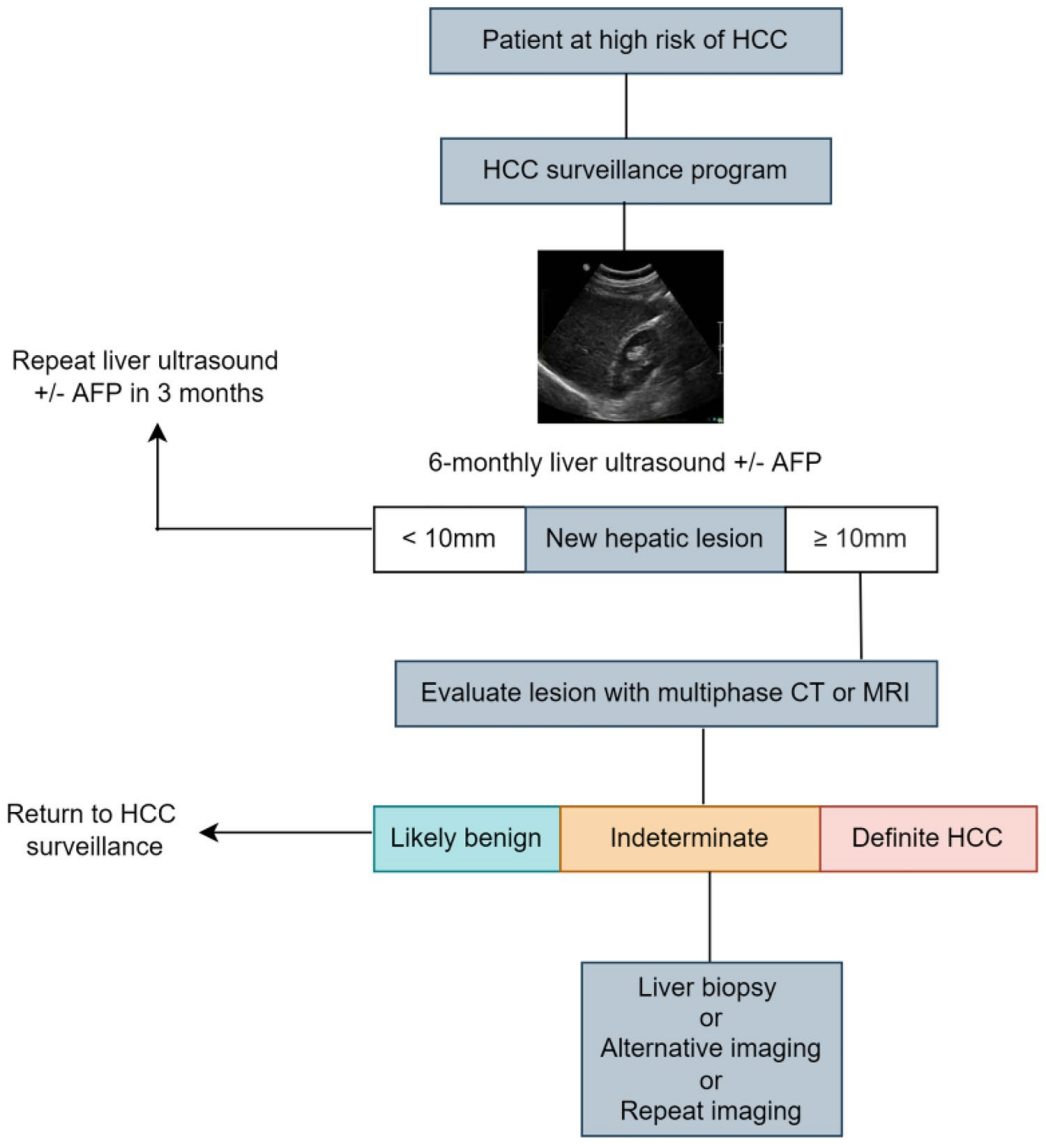
HCC is most common type of primary liver cancer. Alpha-fetoprotein (AFP) is one of the most widely used biomarkers for HCC screening, diagnosis, and prognosis of liver diseases

There is a considerable false positive rate with CT or MRI that triggers further cost-ineffective investigations [24]. These imaging modalities also involve the use of contrast and repeated surveillance would result in accumulated exposure to radiation; and incur higher costs. Therefore, CT and MRI are not recommended for routine surveillance. Contrast-enhanced ultrasound (CEUS) in the delayed phase can be used to detect HCC. A recent meta-analysis showed pooled sensitivity and specificity of CEUS for the diagnosis of HCC at 85% and 91% respectively, with these values being almost comparable with MRI and CT for HCC nodules larger than 2 cm. However its use in surveillance has not been validated and is therefore, currently not recommended. It is important to note that CEUS only allows for one or a limited number of identified nodules as it cannot image the entire liver during the multiple phases of contrast administration [27, 33]. In Fig. 1, the diagnostic workflow of HCC is shown.

Alfa faeto protein (AFP) as a tumour marker for HCC has insufficient sensitivity and specificity for tumour detection when used alone. This is because fluctuating levels of AFP in cirrhotic patients can not only indicate HCC development but may also reflect exacerbation of underlying liver disease or flares of HBV or HCV infection. Also, only a small proportion of tumours at an early stage (10-20%) present with elevated AFP. However, when combined with US surveillance, AFP serum levels’ sensitivity for diagnosing early-stage HCC is significantly higher, 45% when using US alone versus 63% when using US and AFP [34, 35]. The decision to perform a liver biopsy is made on a case-by-case basis. Generally, biopsy is indicated when the imaging-based diagnosis remains inconclusive, but the malignancy is considered probable. As per the EASL Clinical Practice Guidelines, in non-cirrhotic patients, imaging alone is not considered sufficient and tissue assessment is required to establish diagnosis [25]. In Table 1, typical radiological findings of cirrhosis, portal hypertension, and HCC are provided.

**Table 1:** Typical radiological findings of liver disease

**Radiological findings of cirrhosis**	
• Nodular or irregular surface	
• Coarsened liver edge	
• Increased echogenicity	
• Atrophy and multinodular (typically atrophy of right lobe and hypertrophy of carduate or left lobes) - in advanced disease	

**Radiological findings of portal hypertension**	
• Increased portal vein diameter	
• Presence of porto-systematic collateral circulation	
• Reversal of portal vein flow	
• Splenomegaly	
• Ascites	

**Radiological findings of HCC**	
Focal liver lesion with hypervascularity in the late arterial phase and washout on portal venous and/or delayed phases	

## Artificial intelligence, machine learning, and deep learning

The term *Artificial Intelligence* (AI) is an umbrella term and refers to a suite of technologies in which computer systems are programmed to exhibit complex behaviour that would typically require intelligence in humans or animals [36]. The long overarching goal of AI is to enable machines to perform intellectual tasks such as decision making, problem solving, perception and understanding human communication, inspired by the human cognitive function.

Machine Learning (ML), a subset of AI, provides systems the ability to automatically learn and improve from experience without being explicitly programmed [37]. The conventional ML pipeline includes the steps of *pre-processing*, *feature extraction*, *classification*, and *evaluation*. As medical imaging datasets often have variations in characteristics such as *contrast*, *resolution*, *orientation*, *side-markers*, and *noise*, it is important to apply pre-processing techniques to improve the dataset quality. After data cleaning, a relevant region-of-interest (ROI) is selected using either fully-automatic segmentation, semi-automatic segmentation, or manual delineation by experts. After this step, salient features specific to the pattern of a particular medical condition are extracted, in a *feature extraction* step. Once features are extracted, ML algorithms are applied to map extracted features to the target task, such as classification. Overall, ML allows machines to learn from a set of data and subsequently make predictions on a new test data. Applications of ML in medical imaging date back to the early 1980 s when computer-aided detection (CADe) and computer-aided diagnosis (CADx) systems were developed [38]. These CAD(e/x) systems were based on a pre-defined set of explicit parameters, features, or rules developed from expert knowledge. However, one of the major limitations of classical ML systems is the need for handcrafted feature engineering, which is subjective, requires domain expertise and is time-consuming and often brittle.

Deep Learning (DL) [39], a subset of ML, uses multiple layers of neural networks to progressively extract higher-level features from the raw input, overcoming the limitations of hand-crafted feature engineering in classical ML systems. In DL, layers of neural networks are stacked in an hierarchy with increasing complexity and abstraction to obtain high-level representation of the raw data. DL-based models have demonstrated state-of-the-art performance on variety of tasks in various fields such as computer vision, natural language processing, speech, and medical imaging. The success of deep learning is attributed to the availability of large-scale annotated datasets, enhanced computing power with the rise of graphics processing units (GPUs), and novel algorithms and architectures.

In the following subsections, we provide an overview of traditional CAD systems using machine learning and deep learning for diagnosing liver diseases from US images.

### Machine learning based CAD systems

Before the rise of deep learning, classical machine learning based Computer-Aided Detection and Diagnosis (CAD(e/x)) systems involved a pipeline of handcrafted feature extraction and a trainable classifier. The machine learning enabled CAD(e/x) systems assist radiologists in image interpretation, disease detection, segmentation of regions-of-interest (ROI) such as tumours, and statistical analysis of the extracted tumour. In addition to their use in diagnostics, CAD systems are integrated in the clinical workflow to triage and prioritise patients based on urgency, in turn maximising operational performance. A typical CAD system follows a standard pipeline consisting of the following four steps:

1. **Image pre-processing**: Ultrasound (US) images are often of poor quality due to low contrast and presence of speckle noise during image acquisition. The goal of the pre-processing step is to reduce noise, enhance image quality, and standardise the data when it is acquired from multiple sources. Various denoising algorithms such as mean filter, median filter, bilateral filter, and Gaussian filters are applied to remove noise. In order to delineate certain regions, edges are also enhanced using unsharp masking and in the frequency domain. In order to improve contrast, methods such as histogram equalisation and more robust methods such as Contrast Limited Adaptive Histogram Equalisation (CLAHE) are applied. Finally, dataset is normalised using the mean and standard deviation of pixel values. In Table 2, an overview of various pre-processing methods in the reviewed studies to remove noise and improve the image quality is provided. The pre-processing is a critical step in obtaining consistent features and robust model performance.
2. **Image segmentation**: The goal of segmentation is to define the region of interest (ROI) or the volume of interest (VOI) on medical images, that contain the area or volume of the given lesion or structure. Given that normal and abnormal anatomical structures alone do not form the complete image, it is important to segment the image into foreground and background so that ROI can be extracted. ROI selection helps to reduce the computational cost as computing features from ROI is more efficient compared to using the complete image. In the studies reviewed, a variety of segmentation methods are applied, ranging from fully-automatic segmentation algorithms, semi-automatic segmentation algorithms with seed provided by an expert, and manual segmentation for ROI selection. One of the popular semi-automatic methods is *seeded region growing*, in which an expert provides an initial seed point and the algorithm automatically finds the contour by growing over the ROI. Fully-automatic algorithms include *active contour* or *snakes*.
3. **Feature extraction and selection**: The goal of the feature extraction step is to analyse selected ROI for special characteristics that can discriminate disease patterns from normal patterns. It extracts certain characteristic attributes and generates a set of meaningful descriptors from an image. The feature extraction step helps to obtain various quantitative measurements in selected ROI images which helps in decision making with respect to the pathology of a structure or tissue. Common visual features include colour, shape, and texture. Since medical images have homogeneous regions with little colour or intensity variations, shape and texture are more informative features. For US images, the most common characteristics include morphological features, gray-level features, and texture features. Feature extraction can be carried out in the *spatial domain* or *frequency domain*. In Table 3, an overview of various feature extraction algorithms used on most of the articles reviewed in this study is provided. After relevant features are extracted, a subset of the most relevant features are selected using feature selection algorithms. The feature selection step reduces the number of features by removing irrelevant and redundant features, and improves classification performance. The goal of feature selection is to reduce the dimensionality by removing less relevant features, in turn improving the classification accuracy. Common feature selection algorithms used in the reviewed studies include Principal Component Analysis (PCA), Analysis of Variance (ANOVA), and Locality Sensitive Discriminant Analysis (LSDA). In Table 4, an overview of various feature selection algorithms used in the reviewed studies is provided.
4. **Classification**: Classification is the process of categorising items into pre-selected classes or categories of similar type. The items are categorised into classes based on a *similarity * defined by some distance measure. Most of the popular classification methods for CAD of liver diseases include Naive Bayes (NB), K-Nearest Neighbour (KNN), Support Vector Machine (SVM), Artificial Neural Network (ANN), and Discriminant Analysis. Ensemble learning methods such as Random Forests (RF) are also used to leverage the benefit of multiple classifiers and improving classification performance. Table 5 provides an overview of various machine learning classifiers used in the reviewed studies.

**Table 2:** Brief overview of various pre-processing methods to remove noise and enhance the image quality

**Image pre-processing method**	**Description**
Mean filter [40]	The mean filter replaces each pixel value in an image with the mean value of its neighbouring pixels, including itself
Median filter [40]	The median filter replaces each pixel value in an image with the median of neighbouring pixels, including itself
Wiener filter [41]	The Wiener filter is based on statistical properties to filter out the noise that has corrupted the original signal
Bilateral filter [42]	It is a non-linear, edge-preserving, and noise-reducing smoothing filter. It does the spatial averaging without smoothing edges
Gaussian filter [43]	It is linear smoothing filter where the filter (kernel) weights are chosen according to the shape of the Gaussian function
Unsharp masking [44]	The unsharp masking technique sharpens an image by calculating the difference between orignal and its blurred version. It increases the contrast of small details in the magnified texture
Histogram equalisation [44]	It is a technique of adjusting image intensities to enhance contrast. This is achieved by stretching out the most frequent intensities, helping low contrast regions to achieve high contrast. The histogram equalisation method helps to improve the global contrast of the image
Adaptive histogram equalisation [44]	It is adaptive method that computes several histograms, each corresponding to a distinct region of the image, and uses them to redistribute the intensity values of the image. Adaptive histogram equalisation is suitable for improving local contrast in the image
CLAHE [45]	Compared to histogram equalisation and adaptive histogram equalisation that are global contrast enhancement methods, the Contrast Limited Adaptive Histogram Equalisation (CLAHE) performs local contrast enhancement. This has been widely adopted in improving lower contrast in ultrasound imaging

**Table 3:** Brief overview of various feature extraction methods

**Method**	**Description**
First Order Statistics (FOS)	Average gray level (Mean), standard deviation, variance, skewness, kurtosis, uniformity, energy, entropy
Statistical feature matrix (SFM)	Coarseness, Contrast, Periodicity, and Roughness
Law’s Texture Energy Measures	Law’s texture energy measures based on five coefficient vectors to represent level (L), edge (E), spot (S), ripple (R), and wave (W). In total 18 texture features can be extracted
FPS	Radial Sum and Angular Sum of the discrete Fourier transform
Fractal	Hurst exponent, fractal dimension
Gray-Level Difference Statistics (GLDS)	Contrast, differential mean, difference entropy, inverse difference moment, angular second moment
Gray-level Co-occurrence Matrix (GLCM)	Energy, Entropy, Dissimilarity, Contrast, Correlation, Homogeneity, Autocorrelation, Cluster shade, Cluster prominence, Maximum probability, Sum of Squares, Sum Average, Sum Variance, Sum Entropy, Difference Variance, Difference Entropy, Information measure of Correlation, Inverse Difference moment-Normalized
Moment Invariant (MI)	A set of moments invariant to rotation, scaling, and translation derived from second and third normalised central moments
Gradient based features	Mean, Variance, Kurtosis, Skewness, and percentage of pixels with non-zero gradient
Gray-level run-length matrix (GLRLM)	Short run emphasis, Long run emphasis, Gray-lvel non-uniformity, Run-length non-uniformity, Run percentage, Low gray-level run emphasis, High gray-level run emphasis, Short run high gray-level emphasis, Long run low gray-level emphasis, Long run high gray-level emphasis
Gabor Wavelet Transform (GWT)	Mean and standard deviation of Gabor output images obtained by using a set of Gabor wavelets at different scales and orientations
Geometric	Centre of gravity x, Centre of gravity j, Height, Width, Area, Perimeter, Roundness, Euler number, Major axis length, Minor axis length, Orientation, Solidity, Extent, Eccentricity, Convex area, Danielsson factor, Filled area
Frequency-domain	Discrete Cosine Transform (DCT) features, Discrete Wavelet Transform (DWT) features, Wavelet Packet Transform (WPT) features, Curvelet Transform (CT) features, Stationary Wavelet Transform (SWT)
Phase congruency	Variance, contrast, covariance
Gabor texture	Multiple Gabor filters having different frequencies and orientation can be used to extract specific features from an image

**Table 4:** Brief overview of various feature selection algorithms

**Algorithm**	**Description**
Principal Component Analysis (PCA)	It is statistical technique that converts high-dimensional data to low-dimensional data by selecting the most important features that capture maximum information about the dataset. The top most relevant features are selected based on the variance that can explain in the original dataset
Pearson’s Correlation Coefficient	It measures the correlation between features to find out which features are highly correlated and which are not. Based on this analysis, the features that are redundant and do not add value to the final prediction are dropped
Analysis of Variance (ANOVA)	The ANOVA is a statistical method that computes the differences and their variations among the given classes in the data. Based on the statistical analysis, p-value and F-value are computed, based on which significant features are selected
Mutual Information	The mutual information (MI) quantifies the amount of information obtained from one variable through the second variable. Using higher-order statistics calculated using MI, we can select features which can maximise the MI between subset of selected features and the target variable
Fisher score	The Fisher score selects each feature independently based on their scores under the Fisher criterion, providing a subset of most representative features
Locality Sensitive Discriminant Analysis (LSDA)	The LSDA is a feature reduction techniques based on the analysis of studying relationship between data points. The LSDA is effective because it preserves both discriminant and local geometrical structures in the data

**Table 5:** Brief overview of various machine learning algorithms

**Algorithm**	**Description**
Naive Bayes (NB)	The Naive Bayes is a probabilistic classifier based on the Bayes’ Theorem. It predicts the class of a given sample by computing the maximum posterior probability based on the prior probability and the observed likelihood in the training set. The sample is assigned a class with the highest occurring probability
K-Nearest Neighbour (KNN)	The K-Nearest Neighbour classifier is one of the lazy statistical learning algorithms. The training data in KNN algorithm acts as a feature space and during testing, the test sample is compared to all the training samples using a distance metric and label of the training sample having least distance is assigned to the test sample. To improve its robustness, the contributions of the K-neighbours is adopted to decide the label of the test sample
Logistic Regression (LR)	The Logistic Regression is one of the powerful and baseline methods of supervised classification. The ordinary regression is extended to give the probability of outcome between 0 and 1. To use logistic regression as a binary classifier, a threshold is set based on which a sample is discriminated between two classes
Decision Tree (DT)	A decision tree is a tree-based classifier where an internal node represents feature, the branch represents a decision rule, and each leaf node represents the outcome. The decision tree classifier provides the benefits of easy interpretation and efficient handling of outliers
Support Vector Machine (SVM)	The SVM classifier aims to find the optimal hyperplane with the largest margin between positive and negative samples in the high-dimensional feature space. Kernel functions such as Gaussian and Radial Basis Function are used for non-linear mapping of the training data from input space to higher-dimension feature space. The SVM classifier is suitable for complex datasets and shows good generalisation ability on unseen test set
Random Forest (RF)	The RF classifier is an ensemble learning method in which multiple classifiers’ predictions are voted to form the final prediction. In general, ensemble learning methods are robust and provide superior performance given pros and cons of single classifier
Extreme Learning Machine (ELM)	The ELM is a single-layered feed-forward neural network which can be trained in a single pass, making it faster than conventional machine learning algorithms. The ELM has three layers (input, hidden, and output). The weights from input to hidden are randomly initialised and are fixed. During a single pass, the weights from hidden to output layer are learnt by the classifier

### Deep learning

Deep learning (DL) [39] is a subset of machine learning where artificial neural networks, algorithms inspired by the human brain, learn from large amounts of data. DL uses multiple layers to represent data abstractions to build computational models. DL has shown high levels of performance on various complex tasks such as speech recognition [46], machine translation [47], object detection [48], caption generation [49], and visual question answering [50]. Some key deep learning algorithms include convolutional neural networks [51], recurrent neural networks [52], and generative adversarial networks [53]. In the following subsection we provide an overview of the building blocks of a typical convolutional neural network, which is one of the de-facto deep learning algorithms for processing 2D (images) or 3D (volumetric) data.

#### Convolutional neural networks

Convolutional Neural Networks (CNNs) are a special type of deep neural networks that are good at handling two-dimensional data such as images or three-dimensional data such as videos. CNNs have been successfully applied in medical imaging problems such as skin cancer, arrhythmia detection, fundus image segmentation, thoracic disease detection, and lung segmentation. CNNs consists of multiple layers stacked together which use local connections known as local receptive field and weight-sharing for better performance and efficiency. A typical CNN architecture consists of the following layers:

- **Convolutional layer**: The convolutional layer is the core building block of a CNN which uses the convolution operation in place of general matrix multiplication. Its parameters consist of a set of learnable filters, also known as kernels. The main task of the convolutional layer is to detect features within local regions of the input image that are common throughout the dataset and map their appearance to a feature map. The output of each convolutional layer is fed to an activation function to introduce non-linearity. There are a number of activation functions available such as Rectified Linear Unit (ReLU), Sigmoid, etc.
- **Sub-sampling (Pooling) layer**: In CNNs, the sequence of convolutional layer is followed by pooling layer which reduces the spatial size of the input and thus reduce the number of parameters of the network. A pooling layer takes each feature map output from the convolutional layer and down samples it. In other words, the pooling layer summarises a region of neurons in the convolution layer. The most common pooling techniques are *max pooling* and *average pooling*. Max pooling takes the largest value from a patch of the feature map, whereas average pooling takes the average of each patch for the feature map.
- **Activation function**: The activation function refers to the features of activated neurons that can be retained and mapped out by a non-linear function, which can be used to solve non-linear problems. Common activation functions include *sigmoid*, *tanh*, *ReLU*, and *Softmax*. ReLU is one of the widely used activation function as it overcomes the vanishing gradient problem in deep neural networks.
- **Batch normalisation**: Batch normalisation is used to address the issues related to internal covariance shift within feature maps. Internal covariance shift is a change in the distribution of hidden units’ values, which slows down the convergence and requires careful initialisation of parameters. Batch normalisation normalises the distribution of feature maps by setting them to zero mean and unit variance. It also makes the flow of gradients smooth and acts as a form of regularisation, helping the generalisation power of the network.
- **Dropout**: Dropout is a regularisation techniques heavily used in convolutional neural networks. In dropout, some units or connections are randomly dropped (skipped) with a certain probability. Due to multiple connections, a neural network co-adapts by learning non-linear relations. Dropout helps to overcome this co-adaptation by randomly dropping some of the connections or units, preventing the network from overfitting on the training data.
- **Fully connected layer**: In fully connected layers, each neuron from the previous layer is connected to every neuron in the next layer and every value contributes to predicting the class of the test sample. The output of the last fully connected layer is passed through an activation function, generally *softmax*, which outputs the class scores. Fully connected layers are mostly used at the end of the CNN for the classification task.

#### Representative convolutional neural networks

The history of convolutional neural networks dates back to LeNet-5 [54] which was proposed for digit recognition. Due to the lack of computational resources at that time, LeNet usage was limited. The LeNet-5 model uses *tanh* as a non-linear activation function followed by a pooling layer and three fully connected layers. It was the AlexNet [51] model, which made a major breakthrough by drastically reducing the top-5 error rate on the ImageNet challenge compared to the previous shallow networks. Since AlexNet, a series of CNN models have been proposed that have advanced the state of the art steadily on the ImageNet. In AlexNet, tanh is replaced by rectified linear units (ReLU) and the dropout technique is used to selectively ignore units to avoid overfitting of the model. In order to boost predictive performance, Visual Geometry Group at Oxford developed VGG-16 [55]. VGG increased the requirements of memory and computational power because of increased depth of the network with 16 layers combined with convolution and pooling layers. In order to limit memory requirements, various structural or topological decompositions were applied which led to more powerful models such as GoogleNet [56], and Residual Networks (ResNet) [57]. GoogleNet uses an Inception module which computes $1 \times 1$ filters, $3 \times 3$ filters and $5 \times 5$ filters in parallel, but applies bottleneck $1 \times 1$ filters to reduce the number of parameters. Further changes were made to the original Inception module by removing the $5 \times 5$ filters and replacing them with two successive layers of $3 \times 3$ filters, which is called Inception v2. Szegedy et al. [56] released the Inception v3 model where depth, width and number of features are increased systematically by increasing the feature maps before each pooling layer. ResNet was the first network having more than 100 layers using an idea similar to Inception v3. In ResNet, the output of two successive convolutional layers and input bypassing the two layers are combined, which act very similar to a Network-in-Network. ResNet further increases predictive performance by leveraging rich combinations of features, but keeping the computation low. In Table 6, a summary of some of the most common representative CNN models is provided.

**Table 6:** Representative CNN architectures and their high-level description

**CNN architecture**	**Description**
AlexNet [51]	The first CNN model to win the ImageNet challenge in 2012 and brought deep learning revolution. Compared to LeNet, the AlexNet use ReLU activation function, dropout for regularisation, data augmentation during training, and splitting computation on multiple GPUs
VGG [55]	A popular deep CNN model from University of Oxford. The VGG network popularised the idea of using small filter kernels and training the deeper network using pre-training on shallower versions. Two the popular variants of VGG network are: VGG-16 (having 16 layers) and VGG-19 (having 19 layers)
GoogLeNet [56]	Winner of the 2014 ImageNet challenge. This model contains multiple *inception modules*, which provides the idea of multi-scale processing allowing modules to extract features at different levels of detail simultaneously. By stacking multiple CNN layers, model becomes quite complex, yet having less number of model parameters. One of the popular GoogLeNet network is the Inception-v3
ResNet [57]	Winner of the 2015 ImageNet challenge. ResNet networks contains *skip connections* providing information preserving capability by simply copying the activations from lower layers to higher layers. By concatenating and stacking multiple ResNet blocks, it made possible to have much deeper networks, yet having lesser model parameters. Having skip connections in addition to the standard pathway gives network the ability to preserve more information, increasing network’s ability to pick and lose information, learning residuals, and building deeper networks. Major ResNet network variants include ResNet-18, ResNet-50, ResNet-101, and ResNet-152
DenseNet [58]	The DenseNet model uses concatenation of the activations of previous layers to the activation of the current layer. The use of feature maps of all previous layers to the current layer helps to achieve *feature reuse* capability and reducing training parameters. The idea of concatenating activations from previous layers preserve global state, making DenseNets particularly well-suited for smaller datasets, especially medical imaging datasets. One of the important DenseNet model that has been applied by the medical imaging community is the *DenseNet-121* model

#### CNN training strategies

In this section, we highlight various strategies which are helpful in training deep convolution neural networks and improving their performance.

- **Transfer learning**: Transfer learning [59] refers to the ability to share and transfer knowledge from a source task to the target task. Convolutional neural networks learn features in an hierarchical manner, whereby early layers learn generic image features such as edges and corners, whereas later layers learn features specific to the dataset. Given that it is challenging to obtain large-scale annotated datasets in the medical domain due to cost and time constraints, transfer learning helps to leverage the learning of models trained on large-scale datasets such as the ImageNet [60].
- **Data augmentation**: Current state-of-the-art CNNs need large-scale annotated data to train in a supervised manner. Given the complexity of CNN models, it is easy for them to overfit on small size medical imaging datasets. Data augmentation [61] is a technique to generate synthetic data, for example by applying different affine transformations such as rotation, scaling, translation, flipping, and adding noise. Data augmentation not only increases the dataset size during training, but also adds diversity to the data, making the model robust on unseen data.

## Evaluation measures

For evaluating any model, precision, recall, F1-measure, and accuracy scores are computed using the confusion matrix.

- **True Positive (TP)**: If a person having Cirrhosis is detected as Cirrhosis
- **True Negative (TN)**: If a person not having Cirrhosis is correctly detected as non having Cirrhosis
- **False Positive (FP)**: If a healthy person is detected positive for having Cirrhosis
- **False Negative (FN)**: If a person having Cirrhosis is detected as a healthy one.

- **Precision** calculates the fraction of correct positive detection of Cirrhosis.
- **Recall** measures how good all the positives are, which depends on the percentage of total relevant cases correctly classified by the model. It is also called sensitivity.
- **F1-measure** is the harmonic mean between precision and recall.

For a binary classification task, the confusion matrix is a $2 \times 2$ table reporting four primary parameters known as False Positives (FP), False Negative (FN), True Positives (TP), and True Negatives (TN).

1 $$\begin{aligned} \text {Accuracy} = \frac{\text {TP} + \text {TN}}{\text {TP} + \text {FP} + \text {TN} + \text {FN}} \end{aligned}$$

2 $$\begin{aligned} \text {Precision} = \frac{\text {TP}}{\text {TP} + \text {FP}} \end{aligned}$$

3 $$\begin{aligned} \text {Recall} = \frac{\text {TP}}{\text {TP} + \text {FN}} \end{aligned}$$

4 $$\begin{aligned} \text {F-measure} = 2 \times \frac{\text {Precision} \times \text {Recall}}{\text {Precision} + \text {Recall}} \end{aligned}$$

Receiver Operating Curve (ROC) is a 2D graphical plot between the True Positive Rate (Sensitivity) and the False Positive Rate (Specificity). The ROC represents the trade-off between sensitivity and specificity. The Area Under the ROC Curve (AUC) represents a measure of how well a model can discriminate between patients with liver diseases and a healthy group of individuals.

## Contributions

This article aims to characterise diagnosis, staging, and surveillance of liver diseases using medical imaging, machine learning and deep learning techniques through a methodical review of the literature. We seek to answer the following research questions:

1. Methods $\longrightarrow$ What AI methods are being applied to the diagnosis and staging of liver diseases using ultrasound imaging?
2. Datasets $\longrightarrow$ What are different sources of publicly available datasets?
3. Scope $\longrightarrow$ What types of problems are addressed and solved using AI in diagnosing liver diseases?
4. Performance $\longrightarrow$ How well do AI techniques including machine learning and deep learning perform in terms of diagnostic accuracy?

To answer these questions and draw our insights, we methodically studied 77 articles from a variety of publication venues, mostly published between January 2010 to December 2021. There has been surveys related to liver diseases [62–67]. The survey by [62] mostly focused on diffuse liver diseases and cover only conventional CAD systems. [63] focused on radiographic features under different medical imaging modalities for diagnosing liver diseases. Similar to [62, 64, 65] focused on conventional ML pipeline for diagnosing liver lesions using US imaging. Although [66] provided details about both machine learning and deep learning models for diagnosing liver diseases using US imaging, they did not follow a systematic approach. In Table 7, current study is compared to the existing surveys. The current study provides a more detailed and systematic approach of the current state-of-the-art ML and DL approaches for diagnosing liver diseases. We also provide details about the datasets, methods of severity scoring, and professional societies guidelines. We close the review by discussing the limitations of existing studies and noting future research directions to further improve diagnostic performance, expediting clinical workflow, augmenting clinicians in their decision making, and reducing healthcare cost.

**Table 7:** Comparison of review articles related to our survey paper, with their methods and scope

Study	Focus	PRISMA	Methods	Datasets	Data
[62]	Diffuse liver diseases	✗	ML	✗	US
[63]	Liver fibrosis, cirrhosis, and cirrhosis-related nodules	✗	✗	✗	US;MR;CT
[64]	Liver cancer	✗	ML	✗	US
[65]	Applications of Ultrasound imaging	✗	ML	✗	US
[66]	Applications of Ultrasound imaging	✗	ML;DL	✗	US
[67]	Chronic liver diseases	✓	ML	✗	US;CT;MR;ES
This Study	Liver diseases	✓	ML;DL	✓	US

The review is structured as follows:

- Sect. 7 provides search strategy in terms of selected databases, inclusion and exclusion criteria, and keywords related to search query.
- Sect. 8 provides a systematic review of diagnosing liver diseases using ultrasound imaging.
- Sect. 9 provides an overview of various public datasets for the diagnosis of liver diseases.
- Sect. 10 provides current limitations and future research directions.

## Search strategy

We followed the Preferred Reporting Items for Systematic Reviews and Meta-Analyses (PRISMA) guidelines [68] to perform our review.

### Data sources and search queries

We conducted a comprehensive search to identify potentially all relevant publications on the application of AI including machine learning and deep learning to the diagnosis of liver diseases using medical imaging. The Web of Science, Scopus, IEEE Xplore, and the ACM digital library were queried for articles indexed from Jan 2010 up to 31 December 2021. We included articles written in English and excluded those in the form of editorial, erratum, letter, note, or comment. In Table 8, our *inclusion* and *exclusion* criteria are provided. We first identified keywords and their associations to form our search query. For ease of search, we divided our keywords based on four main concepts. The first concept refers to keywords related to liver diseases such as *chronic liver disease(s)*, *acute liver disease(s)*, *liver lesion(s)*, *nonalcoholic fatty liver disease*, and *hepatocellular carcinoma*. The first row in Table 9 shows all keywords relevant to the first concept. The second concept relates to various tasks such as *classification*, *detection*, *segmentation*, and *staging* of liver diseases. The second row in Table 9 shows all keywords for tasks relevant to the second concept. The third concept relates to various imaging modalities by which liver diseases are diagnosed. These include ultrasound, contrast-enhanced ultrasound, computed tomography, and magnetic resonance imaging. The third row in Table 9 shows various keywords and their abbreviations for concepts related to imaging modalities. The fourth concept belongs to keywords related to computer applications such as *computer-aided diagnosis*, *machine learning*, and *deep learning*. For each concept we included associated keywords as well as their abbreviations to make the search criteria complete. The final query is the *logical AND* of all the four concepts. A complete picture of concepts,keywords related to each concept, and the final search query on databases is presented in Table 9.

**Table 8:** List of inclusion and exclusion criteria

**S.No.**	**Inclusion Criteria**
1	Study must be published between January 2010 and December 2021
2	Study must be peer-reviewed journal articles or conference proceedings and written in English
3	Study should have clinical focus on diagnosis of liver diseases using computational techniques
4	Study must have used ultrasound as medical imaging modality
5	Technical studies diagnosing multiple diseases including liver are also considered
6	Study should have performed automated diagnosis of liver diseases using computer applications such as computer vision, machine learning, and deep learning
7	Study must have evaluated the performance of the proposed system using standard evaluation metrics

**S.No.**	**Exclusion Criteria**
1	Study should not be systematic reviews, meta-analysis, and survey papers
2	Study should not focus on diagnosing liver diseases using other imaging modalities such as Computed Tomography (CT), Magnetic Resonance Imaging (MRI), serum biomarkers, liver biopsy, Magnetic Resonance Imaging derived Proton Density Fat Fraction (MRI-PDFF), etc
3	Studies not having technical contribution such as white papers, cases studies, letters, abstracts only

**Table 9:** Search query related to four main concepts which are combined to formulate final query for four databases

**Concepts**	**Keywords**
Concept 1: Keywords related to diagnosis	chronic liver disease(s) OR acute liver disease(s) OR focal liver disease(s) OR diffuse liver disease(s) OR liver lesion(s) OR hepatic disease(s) OR fibrosis OR steatosis OR fatty liver disease OR nonalcoholic steatohepatitis OR NASH OR nonalcoholic fatty liver disease OR NAFLD OR hepatocellular carcinoma OR HCC
Concept 2: Keyword related to tasks	classification OR detection OR localization OR segmentation OR registration OR tracking OR temporal analysis OR severity scoring
Concept 3: Keywords related to imaging modalities	ultrasound OR contrast-enhanced ultrasound OR CEUS OR computed tomography OR CT OR magnetic resonance imaging OR MRI
Concept 4: Keywords related to computer applications	computer-aided OR computer-aided detection OR computer-aided diagnosis OR automated analysis OR artificial intelligence OR machine learning OR deep learning OR deep neural network OR convolutional neural network OR cnn OR dnn OR deep-cnn
**Search query**	(**Concept 1**) **AND** (**Concept 2**) **AND** (**Concept 3**) **AND** (**Concept 4**)

### Article selection

**Fig. 2:**
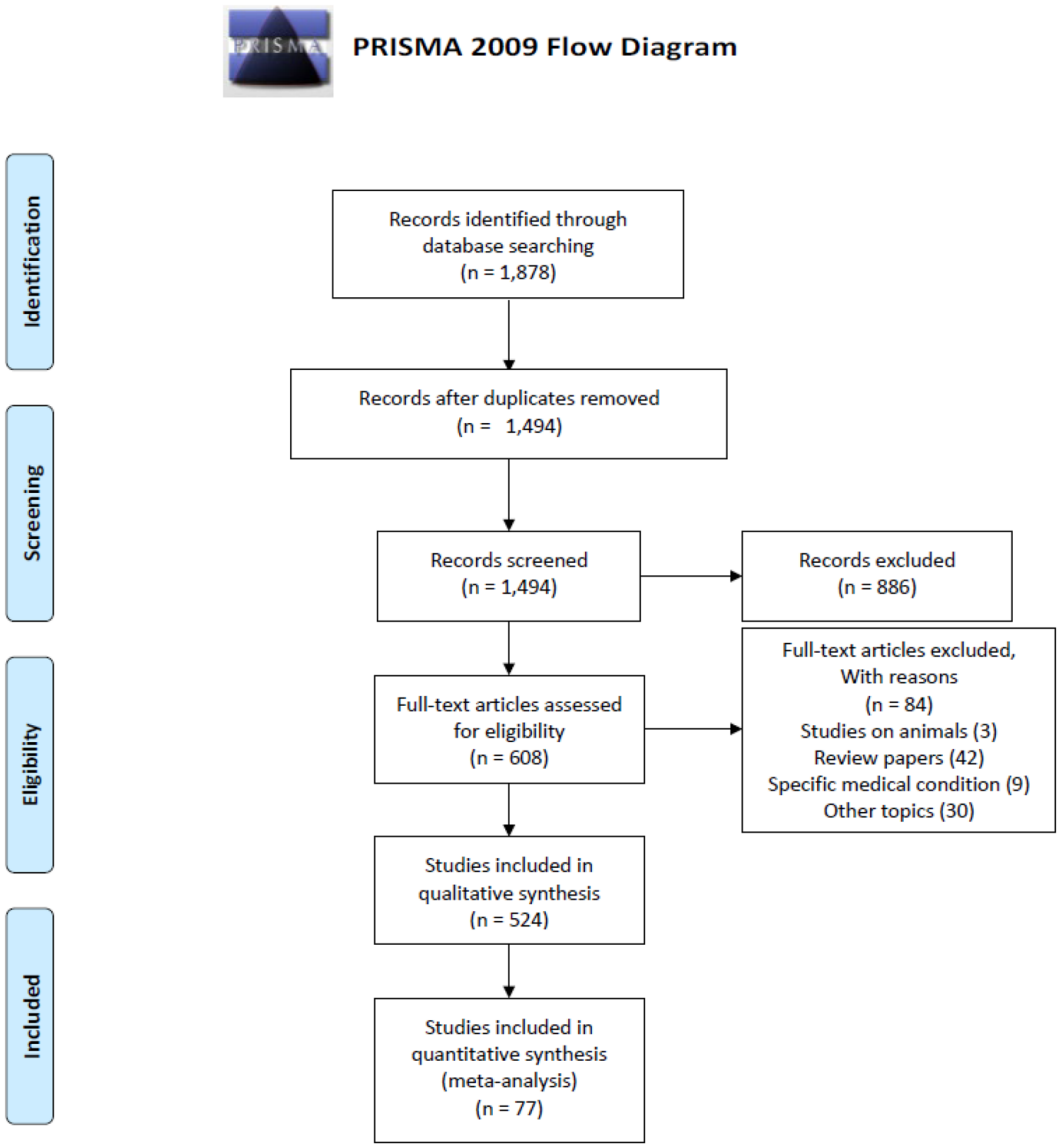
PRISMA flowchart for including articles in our study. The flow diagram depicts the flow of information through the different phases of the methodical review. It maps out the number of studies identified, included and excluded, and the reasons for exclusions

The search query retrieved in total 1,878 studies (Web of Science: 455, Scopus: 543, IEEE Xplore: 786, and ACM digital library: 94). We created an EndNote library for our screening process. We first used “Find Duplicates" function in EndNote to find any potential duplicate studies. The software highlighted 356 duplicate studies which we removed, with a total of 1,700 studies left. Given the limited functionality of the in-built EndNote function for finding duplicates, we then manually removed duplicates. After the manual duplicate removal step, we were left with a total of 1,494 studies. We started our first screening step based on article *title* and *abstract*. We found that there were many studies which are for other human organs but using one of the imaging modalities for diagnosis. After our first screening, we were left with 608 relevant studies. In the second screening step, we read the full-texts of articles and found studies on animals (3), other topic (30), review papers (42), and studies focusing on specific medical condition (9). After excluding all these studies, we were left with a total of 524 studies after our second screening. In the third screening step, we separated studies based on imaging modalities. Out of 524 studies, 243 studies belonged to CT, 81 studies to MRI, and 147 studies to US. However, 53 studies used liver biopsy, blood bio-marker, urine bio-marker, and diffraction enhanced imaging for diagnosing liver diseases, In the final stage of our screening, we reviewed 147 liver US studies and assessed the quality based on the questions in our quality assessment given in Table 10. After the final quality assessment, we were left with a total of 62 studies. During our detailed review, we also went through the references of these 62 studies and found a few relevant studies which our search query was unable to find, to obtain a total of 77 studies. We have reviewed the 77 studies in detail and provide a detailed discussion of the applications, methodology and results. In Fig. 2, the flowchart of our complete article selection process following the PRISMA guidelines is shown. Finally, we conducted a methodical review and qualitative analysis of the 77 studies in accordance with our inclusion and exclusion criteria. The article selection was performed by the first author (S.S.) and was agreed by all other authors of this article.

Due to the heterogeneity and multidisciplinary nature of the included studies, a formal meta-analysis is not possible. We did, however, visually determine overall performance by representing different performance metrics including sensitivity, specificity, F1-score, and receiver operating characteristic (ROC) curves, which are presented later.

**Table 10:** Quality assessment questions

**S.No.**	**Question**
1	Are research objectives clearly defined?
2	Is research methodology well-defined?
3	Is the train and test data source clearly defined?
4	Are the data pre-processing techniques clearly defined and their selection justified?
5	Are the feature extraction or feature engineering techniques clearly described and justified?
6	Are the learning algorithms clearly described?
7	Does the study perform the comparison with the existing baseline models?
8	Is the performance of the proposed system evaluated and results properly interpreted and discussed?
9	Does the conclusion reflect the research findings?

## Review of studies on the diagnosis and staging of liver diseases

**Fig. 3:**
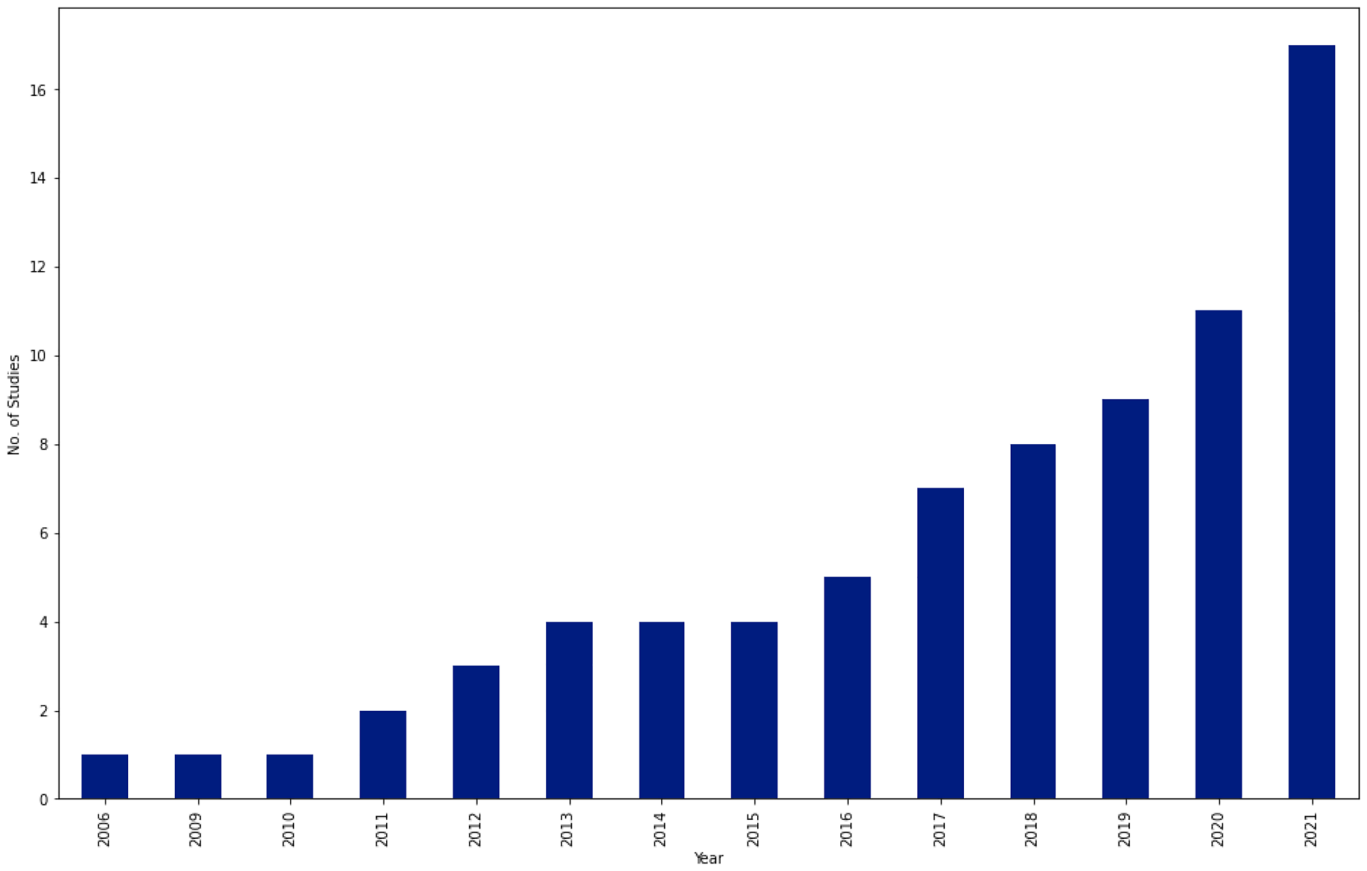
Number of studies per year. In total there were 77 selected studies that meet our selection criteria

**Fig. 4:**
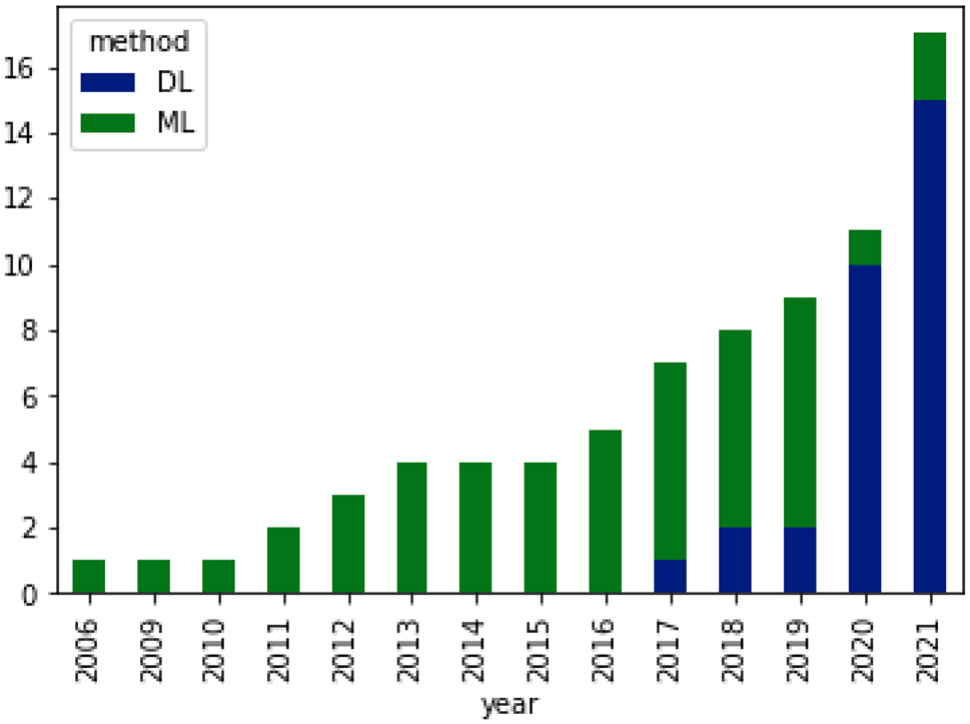
Number of studies applying ML (Machine Learning) and DL (Deep Learning) methods

**Fig. 5:**
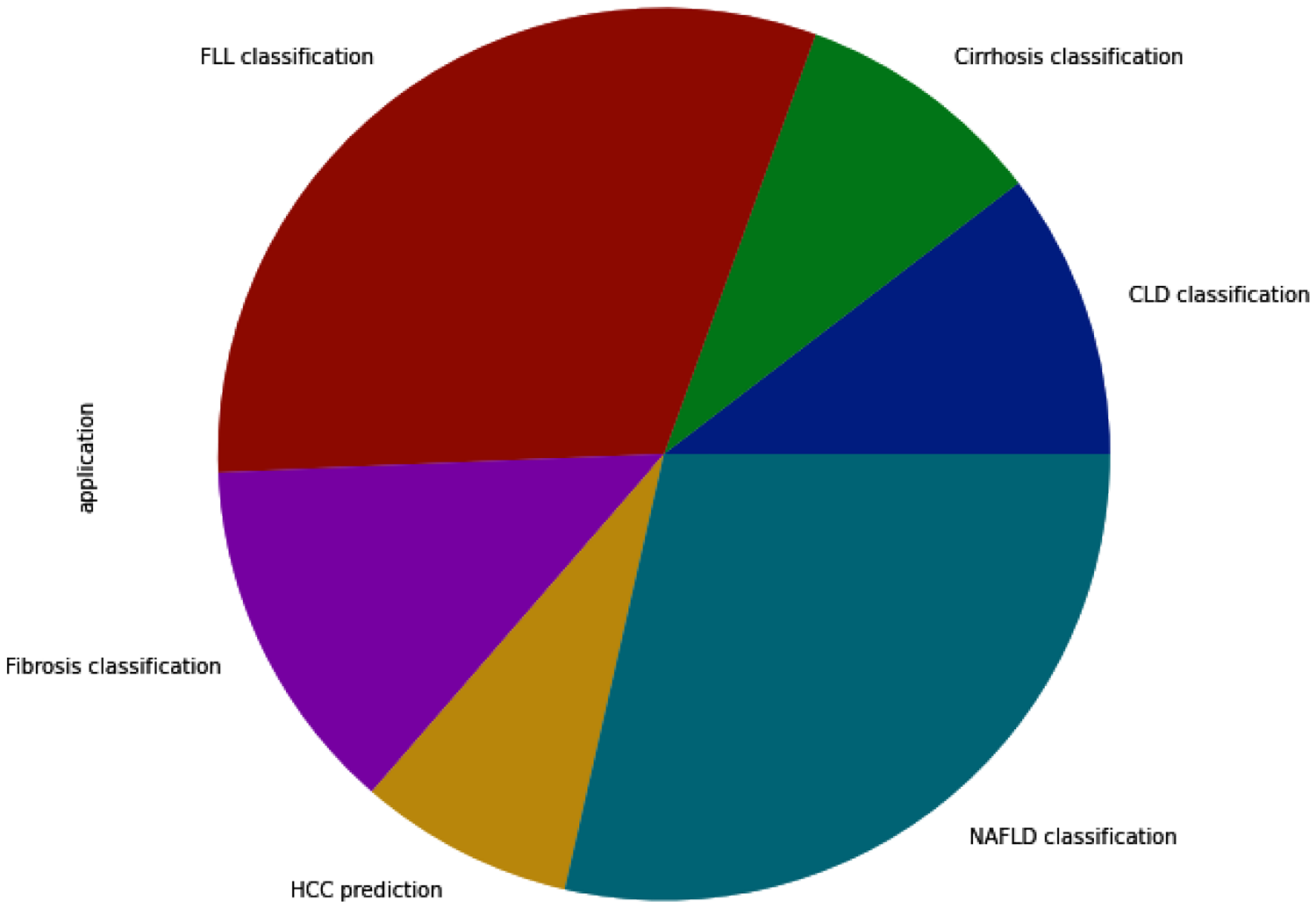
Distribution of studies application wise. NAFLD classification (n=22/28.57%), CLD Classification (n=8/10.38%), Fibrosis classification (n=10/12.98%), Cirrhosis classification (n=7/9.09%), FLL classification (n=24/31.16%), and HCC prediction (n=6/7.79%)

In this section, we review selected studies based on the disease of interest. In Table 11, a summary on studies for fibrosis classification or staging is provided. Most of these studies extracted textural features and applied conventional machine learning algorithms for classification. A few of studies [69, 70] performed fusion of multiple ultrasound modalities to improve diagnostic performance on fibrosis staging. In Table 12, a summary of studies on cirrhosis classification is provided. The focus is on separating normal cases from Cirrhosis. In terms of methods, studies have applied both conventional machine learning and deep learning methods. Table  13 provides summary of studies focusing on nonalcoholic fatty liver disease diagnosis. Most of the studies applied a combination of texture feature extraction algorithms and conventional ML classifiers. Table 14 provides summary of classification of various chronic liver diseases. In this setup, studies focus on three class classification (Normal vs. NAFLD vs. Cirrhosis) or four class classification (Normal vs. Steatosis vs. Fibrosis vs. Cirrhosis). One study [71] performed six class classification with six labels namely, normal, steatosis, chronic hepatitis without cirrhosis, compensated cirrhosis, decompensated cirrhosis, and HCC. In Fig. 3, an year-wise number of studies is shown. The plot shows an increasing trend in the number of studies over years. In Fig. 4, we provide number of studies applying ML or DL methods for diagnosing liver diseases. The plot shows that machine learning was a de-facto choice before 2017. However, with the rise of deep learning, there has been a sharp increase in the number of studies using deep learning methods. In Fig. 5, the distribution of studies is given based on various applications. Of the 77 studies covered in this review, the distribution is as: *Fibrosis classification* (n=10), *Cirrhosis classification* (n=7), *NAFLD* (n=22), *CLD* (n=8), *FLL* (n=24), and *HCC diagnosis and prognosis (n=6)*. Most of these studies focus on the classification problem considering one disease versus rest of the diseases.

The results of studies in Table 14 show that as the number of classes increase, the performance of models degrade. This is due to the correlation between diseases and overlapping biomarkers of diseases. In Table 15, a summary of studies focusing on different liver lesions and hepatocellular carcinoma is presented. A major observation is that most of the studies focused on small private (in-house) datasets containing only a few samples of liver lesions such as hemangioma (HEM) or metastases (MET). The studies provide quite high classification performance in terms of accuracy scores. However, accuracy is not a robust metric especially when working with imbalanced datasets. Finally, Table 16 provides summary of studies focusing on HCC prognosis. Most of these studies focused on patient survival analysis. Few of the recent studies showed that CNNs outperforms classical ML algorithms for HCC diagnosis and prognosis.

**Table 11:** Summary of studies on Fibrosis classification or staging using liver US images

**Study**	**Dataset(s)**	**Data Pre-processing**	**Feature extraction and Selection**	**Learning method(s)**	**Results**	**Main finding(s)**
[72]	55 patients having fibrosis stages (F1: 17; F2: 12; F3: 5; F4: 21)	ROI selection	11 elastic parameters obtained from RTE by PCA. Blood biomarkers as additional features	Spearman’s correlation coefficient	AUC: 0.93	RTE images along with histology results is effective in diagnosing liver fibrosis
[73]	279 US images	Data augmentation	Features are extracted from VGGNet fc7 layer	RF, SVM, GBDT, MLP, FCNet	Accuracy96.06%	Features extracted from pretrained CNN model with FCNet as classifier outperforms conventional ML classifiers
[74]	513 patients US images	Encoding diagnostic and demographic parameters	Textural features	NB, RF, KNN, SVM	Sensitivity (77.0%), and Specificity (77.3%)	ML classifiers showed superior performance for liver fibrosis staging than liver fibrosis index (LFI) method
[75]	144 patients suffering from Hepatitis B		Multi-parametric features; Spearman’s correlation coefficient for feature selection	DT, LR, ANN, RF, SVM	Mean AUC of 0.85	Multi-parametric features improves staging of liver fibrosis compared to mono or dual modalities
[76]	229 patients providing 10 US images each		Multi-stream feature extraction using VGG-16	ANN	Overall accuracy: 65.6%	An indicator-guided ML framework provides better results for fibrosis diagnosis
[70]	466 patients (Normal: 64; Fibrosis: 401)	Automatic ROI selection made on gray-scale and 2D SWE images	Inception-V3 model to extract 4096-dimensional embeddings from B-mode and SWE images	Softmax classifier	AUC of 0.950, 0.932, and 0.930 achieved for classifying fibrosis of stage S4, S3, and S2, respectively	Combination of gray-scale images and 2D SWE images for better results than a single modality
[77]	13,608 US images	Liver US images without a focal hepatic lesion were used		VGGNet	Accuracy of 4-class classification: Internal test data (83.5%) and external test data (76.4%)	CNNs achieved at par performance to that of radiologists in determining METAVIR score using US images
[78]	157 subjects having fibrosis stages (F0: 44; F1: 31; F2: 35; F3: 20; F4: 27)	Speckle noise reduction, ROI selection and data augmentation	Image features extracted along Glisson’s line	MLNN and CNN	Accuracy (five class): 84.38%	Focusing on region along the Glisson’s line improves fibrosis classification
[79]	550 B-mode US images from 55 participants (NAFLD: 38; Normal: 17)	ROI selection and data augmentation	Features extracted from pre-trained ResNet-V2, GoogleNet, AlexNet, and ResNet-101	SVM	Overall accuracy: 98.64%, Sensitivity: 97.20%, and Specificity: 100%	Concatenation of features from diverse CNN models is helpful in improving diagnostic performance of NAFLD classification
[69]	214 subjects providing US images			Multi-modal fusion network with active learning	AUC: 0.897 and accuracy: 70.59%	Fusion of multiple US modalities along with active learning demonstrated better performance
[80]	286 US images	ROI selection, data augmentation		CNN framework	Accuracy: 95.66%	Multi-scale information and local attention mechanism can provide accurate liver fibrosis classification
[81]	700 US images	Contour detection and CLAHE	CNN features	Softmax, SVM	Accuracy: 98.59%	Deep features extracted by CNN are robust for fibrosis classification
[82]	640 US images		Textural features along with patient’s age and gender	AlexNet, VGG-16, VGG-19, GoogleNet	Accuracy: 95.29%	Combination of patient characteristics along with image features improved classification accuracy

*SVM* Support Vector Machine, *RF* Random Forest, *LR* Logistic Regression, *NB* Naïve Bayes, *ANN* Artificial Neural Network, *KNN* K-Nearest Neighbour, *PNN* Probabilistic Neural Network, *DT* Decision Tree, *ELM* Extreme Learning Machine, *CNN* Convolutional Neural Network, *ROI* Region-of-Interest, *US* Ultrasound, *CEUS* Contrast Enhanced Ultrasound, *SWE* Shear Wave Elastography, *HCC* Hepatocellular Carcinoma, *NAFLD* Nonalcoholic Fatty Liver Disease, *HEM* Hemangioma, *MET* Metastasis, *FLL* Focal Liver Lesions

**Table 12:** Summary of studies on Cirrhosis classification using liver US images

**Study**	**Dataset(s)**	**Data Pre-processing**	**Feature extraction and Selection**	**Learning method(s)**	**Results**	**Main finding(s)**
[83]	60 US images (Normal: 20; Cirrhosis: 40)	ROI selection	Uniform LBP and GLCM for feature extraction, PCA for feature selection	SVM	Overall accuracy: 87.0%	Uniform-LBP features can better describe the cirrhotic features in US images
[84]	91 US images (Normal: 44; Cirrhosis: 47)	Data normalisation	Liver capsule detection using sliding window and dynamic programming based linking	CNN	Mean accuracy of 89.2%, AUC of 0.968	Liver capsule detection based features are effective in diagnosing cirrhosis from liver US images
[85]	147 US images (Normal: 75; Cirrhosis: 72)	Manual ROI selection	Textural features. The correlation-based filter (CFS) for feature selection	KNN, SVM	Overall accuracy: 99.31%	Combination of Wavelet and Curvelet features provided superior performance than other feature extraction methods
[86]	110 US images (30 Normal and 80 Cirrhosis)	Manual ROI selection	Intensity difference technique	SVM	Overall accuracy of 98.18%, sensitivity of 98.75%, and specificity of 96.67%	Intensity difference technique provides discriminative features for cirrhosis and normal liver
[87]	500 US images (Normal: 200; Cirrhosis: 300)	Data augmentation	2048-dimensional CNN embeddings from each ROI	ANN	Overall accuracy: 68.1%	CNN using transfer learning and data augmentation outperforms conventional methods
[88]	681 US images having B-mode and color doppler data	Automatic ROI selection		LiverTL having pre-trained VGG-16 as the backbone model	Overall AUC of 0.948	Transfer learning applied on ROI images improves cirrhosis diagnosis
[89]	69 patients providing B-mode US images	Radiologist delineated boundaries of liver surface		Pre-trained GoogLeNet	AUC score of 0.992	Liver shape information acts as a strong indicator for cirrhosis diagnosis
[90]	189 US images (70 normal, 94 steatotic, 25 cirrhotic)	ROI selection	Textural features	Accuracy: 85.3%	Feature selection and adding liver length as parameter improved classification accuracy	

*SVM* Support Vector Machine, *RF* Random Forest, LR Logistic Regression, *NB* Naïve Bayes, *ANN* Artificial Neural Network, *KNN* K-Nearest Neighbour, *PNN* Probabilistic Neural Network, *DT* Decision Tree, *ELM* Extreme Learning Machine, *CNN* Convolutional Neural Network, *ROI* Region-of-Interest, *US* Ultrasound, *CEUS* Contrast Enhanced Ultrasound, *SWE* Shear Wave Elastography, *HCC* Hepatocellular Carcinoma, *NAFLD* Nonalcoholic Fatty Liver Disease, *HEM* Hemangioma, *MET* Metastasis, *FLL* Focal Liver Lesions

**Table 13:** Summary of studies on NAFLD classification using liver US images

**Study**	**Dataset(s)**	**Data Pre-processing**	**Feature extraction and Selection**	**Learning method(s)**	**Results**	**Main finding(s)**
[91]	20 US images (Normal: 10; Fatty: 10)	Manual ROI selection	Textural features	Bayesian classifier	Overall accuracy: 95%	RF and speckle images can accurately capture textural features relevant for diagnosis of fatty liver disease
[92]	100 US images (Normal: 42; NAFLD: 58)	ROI selection	Textural features	DT, Fuzzy classifier	Overall accuracy: 93.3%	Combination of texture and DWT-based features improves diagnostic accuracy of the model
[93]	US images (NAFLD: 30; Normal: 30; Heterogeneous: 19)	Manual ROI selection	Wavelet Packet Transform (WPT)	SVM	Accuracy (3-class classification): 95.4%	Multi-scale analysis using WPT provides performance is suitable in assisting experts for accurate FLD diagnosis
[94]	180 US images (Normal: 80; Fatty: 100)	Manual ROI selection	Textural features, LDA for feature selection	Linear model with information fusion	Overall accuracy: 95.0%	Information fusion based classification can provide superior performance for NAFLD diagnosis
[95]	53 US images (Normal: 12; Mild: 14; Moderate: 14; Severe fatty liver: 13)	Liver region-of-interest (LROIs) and diaphragm region-of-interest (DROI) from each US image	Textural features; Differential evolution feature selection (DEFS) algorithm	SVM	Overall classification accuracy: 84.9±3.2	Features extracted from liver parenchyma (LROIs) along with features extracted from diagphragm ROI (DROIs) helps to improve overall classification performance
[96]	394 subjects providing US images, radio-frequency data		Textural, backscattering, and attenuation features	Statistical analysis	AUC of 0.73 for NAFLD and 0.81 for severe NAFLD on test set	Quantitative diagnostic index can distinguish mild and severe NAFLD from normal liver
[97]	100 US images (Normal: 50; Fatty: 50)	Manual ROI selection	GIST descriptors as features. Marginal Fisher Analysis for dimensionality reduction	KNN, DT, SVM, AdaBoost, PNN	Accuracy: 98%, Sensitivity: 96%, Specificity: 100%, AUC: 0.9674	GIST features are significant characteristics of fatty liver disease
[98]	100 US images (Normal: 50; Fatty: 50)	Image normalisation, CLAHE	Radon Transform (RT) and 2D-DCT coefficients as features. LSDA for dimensionality reduction	DT, KNN, PNN, SVM, AdaBoost, Fuzzy Sugeno classifier	Accuracy: 100%, Sensitivity: 100%, Specificity: 100%	Combination of RT and 2D-DCT features are good discriminators for fatty liver disease
[99]	63 subjects (NAFLD: 36; Normal:27)	ROI selection	Textural and Gabor directional features	SVM, ELM	Accuracy: ELM (97.75%) vs SVM (89.01%); AUC: ELM (0.97) vs SVM (0.91)	ELM based classifier provided superior results compared to SVM for characterisation and stratification of NAFLD
[100]	650 patients (Normal: 196; Grade-I: 173; Grade-II: 157; Grade-III: 124)	Images cropped to form 1,000 texture patches	Textural features, Gabor filter, and curvelet transform	KNN, SVM	Overall accuracy: 96.9%	The curvelet transform features in combination with SVM classifier gave highest accuracy in diagnosing various grades of fatty liver disease
[101]	90 US images (Normal: 45; Fatty: 50)	Manual ROI selection by radiologist	Textural and Fractal features. Mutual Information (MI) for feature selection	SVM, KNN, DT, AdaBoost	Accuracy: 95.55%, Sensitivity: 97.77%	Mutual information feature selection is an effective technique to select best features for diagnosing fatty liver disease
[102]	63 subjects (NAFLD: 36; Normal: 27)	ROI selection	Textural features	SVM, ELM, and CNN (Inception model)	Accuracy: SVM (82%), ELM (92%), and CNN (100%)	CNN model outperformed ML-based system for ultrasound tissue characterisation
[103]	55 subjects (NAFLD: 38; Normal: 17) providing 550 US images	Data cleaning, images resizing	GLCM and CNN-based features	SVM	AUC: CNN-based (0.977), HI-based (0.959), and GLCM-based (0.892)	CNN based features from B-mode US images can be used for NAFLD diagnosis
[104]	57 US images (Normal: 25; Fatty: 32)	Manual ROI selection	Textural features. PCA for dimensionality reduction	KNN, MLP-kernel SVM	Overall accuracy: 98.78%	Wavelet features are computationally efficient and manufacturer independent, and are suitable for NAFLD diagnosis
[105]	1,000 images (Normal:250; Mild:250; Moderate:250; Severe:250)	No pre-processing applied	Textural and transfer scattering coefficients (SC) features	KNN, SVM, EKNN	Overall accuracy: 98.8%	Compressed transfer SC features proved to be effective in representing the texture of fatty liver and providing good classification accuracy
[106]	577 subjects (Normal: 200; Fatty liver: 377)	SMOTE method to generate synthetic samples to balance classes	Each variable weighted by the information gain ranking process	RF, LR, ANN, NB	Accuracy: 86.48%, Sensitivity: 87.16%, Specificity: 85.89%	RF classifier showed superior performance compared to NB, ANN, and LR classifiers for NAFLD diagnosis
[107]	204 subjects (Normal: 64; Fatty: 140)	Manual ROI selection		1-D CNN	Accuracy: 96%, Sensitivity: 97%, Specificity: 94%	DL algorithms using RF data can accurately diagnose NAFLD and hepatic fat fraction quantification
[108]	240 subjects (Normal: 106; Mild: 57; Moderate: 67; Severe: 10)	Manual ROI selection		Customised CNN model	AUC: 0.933	DL algorithms showed better diagnostic ability than gray-scale values in moderate and severe NAFLD
[109]	55 subjects (NAFLD: 38; Normal: 17) providing 550 US images	Manual ROI selection, data augmentation	Local phase and radial symmetry features	Multi-scale CNN	Overall accuracy: 97.8%	Local phase-based image enhancement and feature representation helps to improve NAFLD diagnosis
[79]	55 subjects (NAFLD: 38; Normal: 17) providing 550 US images	ROI selection, data augmentation	Features extracted from ResNet-V2, GoogleNet, AlexNet, and ResNet-101	SVM	Overall accuracy: 98.64%, Sensitivity: 97.20%, and Specificity: 100%	Concatenation of features from diverse CNN models is helpful in improving diagnostic performance of NAFLD classification
[110]	2,070 patients providing 21,885 US images	Contrast enhancement and noise removal using CLAHE and Gaussian filter		Various CNN models	AUC scores for mild steatosis vs others: 0.974	DL can predict early stage steatosis level with good performance
[111]	90 subjects	Images resizing, data augmentation and data normalisation		VGG-19	Overall accuracy: 80.1%, precision: 86.2%, and specificity: 80.5%	Multi-view US images and DL can effectively classify fatty liver disease and measure fat fraction values
[112]	300 US images (155 normal and 145 fatty cases)	Multiple ROI selection	Textural features	Genetic Algorithm	Accuracy: 95.71%, F1-score: 95.64%	Ensemble algorithms can improve NAFLD classification accuracy
[113]	1,119 US images from 106 patients			Customised ML model in AutoML	Precision: 88.98%, Recall: 88.24%	AutoML tool has potential for aiding a physician in diagnosing NAFLD on US images
[114]	235 participants providing 245 US images	Manual ROI selection	Textural features	XGBoost, Random Forest, SVM	AUC:0.8	ML model provides at par performance to CAP model

*SVM* Support Vector Machine, *RF* Random Forest, *LR* Logistic Regression, *NB* Naïve Bayes, *ANN* Artificial Neural Network, *KNN* K-Nearest Neighbour, *PNN* Probabilistic Neural Network, *DT* Decision Tree, *ELM* Extreme Learning Machine, *CNN* Convolutional Neural Network, *ROI* Region-of-Interest, *US* Ultrasound, *CEUS* Contrast Enhanced Ultrasound, *SWE* Shear Wave Elastography, *HCC* Hepatocellular Carcinoma, *NAFLD* Nonalcoholic Fatty Liver Disease, *HEM* Hemangioma, *MET* Metastasis, *FLL* Focal Liver Lesions

**Table 14:** Summary of studies on CLD classification using liver US images

**Study**	**Dataset(s)**	**Data Pre-processing**	**Feature extraction and Selection**	**Learning method(s)**	**Results**	**Main finding(s)**
[115]	680 US images (Normal: 200; Fatty: 160; Cirrhosis: 160; Liver cancer: 160)	Two ROIs of 64 x 64 pixels selected	Mean, Variance, Skewness, and kurtosis	ANN	Overall accuracy: 96.125%	Automatic liver tissue can be characterised by liver US images for the diagnosis of liver diseases
[71]	97 US images (Normal: 30; Steatosis: 4; Chronic hepatitis without cirrhosis: 9; Compensated cirrhosis: 35; Decompensated cirrhosis: 35; and HCC: 6)	ROI of 128 x 128 pixels along the medial axis	Acoustic attenuation coefficients, FOS, GLCM	DT, SVM, KNN	Overall accuracy: 73.20% using SVM	Non-invasive methods provide reliable information about the staging of chronic liver diseases
[116]	150 US images (Normal: 50; NAFLD: 50; Cirrhosis: 50)	CCA to generate bounding box around liver region, Image cropping, CLAHE for contrast enhancement	Curvelet Transform for feature extraction, LSDA for feature reduction	DT, SVM, KNN, LDA, QDA, NB	Accuracy: 97.33%, Specificity: 100.0%, Sensitivity: 96.0%	Liver disease index (LDI), one of the factor developed using LSDA coefficients an be used to differentiate fibrosis and cirrhosis disease
[117]	129 US images (Normal: 29; Steatosis: 47; Fibrosis: 42; Cirrhosis: 12)	Coordinates conversion and automatic ROI selection	Textural features both in spatial domain and transform coefficients (FPS, DWT, WPT)	KNN, ANN, SVM	Accuracy: 94.91%; Sensitivity (normal): 100; Sensitivity (steatosis): 100; Sensitivity (fibrosis): 87.5; Sensitivity (cirrhosis): 100	Textural features in combination with hierarchical classification can diagnose liver diseases, avoiding invasive method of liver biopsy
[118]	79 CLD cases (Liver cancer: 44 and liver abscess: 35)	Manual ROI selection	Textural features, Sequential forward selection and sequential backward selection for feature selection	SVM	Overall highest classification accuracy of 89.25%	Features selected by sequential forward selection gave highest classification performance
[119]	279 US images (Normal: 95; Steatosis: 105; Cirrhosis: 79)	Multiple ROIs selected from each US image by an expert	Correlation, homogeneity, entropy, variance, energy, contrast, standard deviation, and run percentage. Fisher discriminant for feature selection	Majority Voting	Accuracy scores: Normal/Steatosis (95%), Normal/Cirrhosis (95.74%), Steatosis/Cirrhosis (94.23%)	A combination of different feature extraction methods along with voting classifier is superior in diagnosis diffuse liver diseases
[120]	216 US images (Normal: 72; Hepatitis: 72; Cirrhosis: 72)	ROI selection, data augmentation		AlexNet, ResNet	Overall accuracy: 86.4%	Fine-tuning pre-trained CNN models improves diagnostic performance
[121]	264 US images (Normal: 128; Diffuse liver disease:: 136)	ROI selection made by radiologist	Textural features	Self-organisation feature maps (SOFM)	Sensitivity: 98.6%, Specificity: 98.2%	Textural features are good discriminators for diagnosing liver pathology

*SVM* Support Vector Machine, *RF* Random Forest, *LR* Logistic Regression, *NB* Naïve Bayes, *ANN* Artificial Neural Network, *KNN* K-Nearest Neighbour, *PNN* Probabilistic Neural Network, *DT* Decision Tree, *ELM* Extreme Learning Machine, *CNN* Convolutional Neural Network, *ROI* Region-of-Interest, *US* Ultrasound, *CEUS* Contrast Enhanced Ultrasound, *SWE* Shear Wave Elastography, *HCC* Hepatocellular Carcinoma, *NAFLD* Nonalcoholic Fatty Liver Disease, *HEM* Hemangioma, *MET* Metastasis, *FLL* Focal Liver Lesions

**Table 15:** Summary of studies on Focal Liver Lesions (FLLs) classification using liver US images

**Study**	**Dataset(s)**	**Data Pre-processing**	**Feature extraction and Selection**	**Learning method(s)**	**Results**	**Main finding(s)**
[122]	450 US images (Liver cancer: 50; Hepatocellular adenoma: 150; HEM: 35; Focal nodular hyperplasia:145; Lipomas: 70)	Manual ROI selection by sonographer	Texture features including enery, contrast, correlation, entropy, and homogeneity	SVM, Fuzzy-SVM	AUC (5-class classification): 0.971±0.012	Combination of GLCM textural features with Fuzzy-SVM classifier provides superior results
[123]	111 B-mode US images (Normal: 16; Cyst: 17; HCC: 15; HEM: 18, MET: 45)	Speckle noise removal and an ROI selection of 25 x 25 pixels	FOS, GLDM, GLRLM, Law’s TEM, and GWT	MLP	Overall accuracy (5-class classification): 86.4%	Two-step neural network classifier training showed superior performance in classifying focal liver lesions from US images
Virmani et al. (2013)	108 US images (Normal: 21; Cyst: 12; HEM: 15; HCC: 28; MET: 32)	Manual ROI selection by radiologist	Texture and Gabor features, PCA for dimensionality reduction	SVM	Overall accuracy: 87.2%	Texture features in combination with PCA and SVM classifier gave better results in diagnosing liver lesions
[124]	51 US images (HCC: 27; MET: 24)	Manual ROI selection by an experienced radiologist of size 32 x 32 pixels	GLCM, GLRLM, FPS, and Law’s TEM. GA-SVM for feature selection	SVM	Overall classification accuracy of 91.6% with sensitivity of 90% and 93.3% for HCC and MET cases	ML-based CAD systems can assist radiologists in diagnosing liver malignancies and facilitating better disease management
[125]	150 US images (Cyst: 50; HEM: 50; Malignancies: 50)	7 ROIs representing echo, morphology, edge, echogenicity, and posterior echo enhancement	GLCM, FOS, algebraic moment invariant (AMI), auto-correlation (AC), Laws’ TEM, and Gabor Wavelet features for each ROI	SVM	Accuracy for Cyst vs. HEM: 93.77%, Cysts vs. Malignancies: 92.13%, HEM vs. Malignancies: 69.33%	Multiple ROIs representing varied characteristics of liver US can provide enhanced and stable classification performance compared to single ROI for each US image
[124]	51 US images (HCC: 27; MET: 24)	Manual ROI selection by an experienced radiologist of size 32 x 32 pixels	GLCM, GLRLM, FPS, and Law’s TEM. GA-SVM for feature selection	SVM	Overall accuracy of 91.6% with sensitivity of 90% and 93.3% for HCC and MET	ML-based CAD systems can assist radiologists in diagnosing liver malignancies and facilitating better disease management
[126]	56 B-mode US images (Normal: 15; Cirrhotic: 16; HCC: 25)	ROI selection by an experienced radiologists of size 32 x 32 pixels	DWT, SWT, and WPT features. GA-SVM for feature selection	SVM	Overall accuracy: 88.8%; Sensitivity: 90.0% for normal and cirrhotic liver and 86.6% for HCC	Non-invasive imaging methodologies can be used for diagnosing liver diseases, in turn avoiding liver biopsies
[127]	108 B-mode liver US images	Two ROIs, inside ROIs and surrounding ROIs by an experienced radiologist	GLCM, GLRLM, FPS, Laws’ TEM, and Gabor features. PCA for feature selection	PCA-NN	Overall 5-class classification accuracy: 95.0%	Incorporating texture ratio features along with texture features computed from surrounding region of the lesion improve diagnosis of FLLs
[128]	60 US images (Normal: 30; Fatty: 10; Cirrhosis: 10; Hepatomegaly: 10)	ROI selection using active snake contour model	Intensity histogram, GLCM, GLRLM, MI, and mixed features	ANN	Overall accuracy: 95%	GLRLM features shows better results for focal liver lesion classification
[129]	26 CEUS videos (HCC: 6; HEM: 10; Abscesses: 4; MET: 3; Localised fat sparings: 3)	Salient frames from each video are selected, image correction techniques applied	Time intensity curves (TIC) features extracted by sparse non-negative matrix factorizations	LDA, KNN, SVM, BPN, Deep Belief Networks (DBN)	Accuracy: 86.36%, Sensitivity: 83.33%, Specificity: 87.50%	Deep Belief Networks trained using TIC features outperforms conventional ML algorithms for focal liver lesions classification
[130]	94 US images (Normal, Cyst, HEM, HCC)	Noise removal using bilateral filtering. Automatic ROI selection	A total of 94 features containing 6 histogram features and 88 GLCM based texture features	KNN, Multi-SVM	Overall accuracy: 96.11%, sensitivity: 97.08%, Specificity: 91.83%	Multi-SVM gave superior results compared to KNN classifier for the staging of focal liver lesions
[131]	52 CEUS video sequences (FNH: 13; HEM: 17; HCC: 16; MET: 6)	Salient frames selected from each video. Liver lesion ROI selection by a radiologist	Time intensity curve (TIC) and morphology features	SVM	Overall accuracy: 90.3%, sensitivity: 93.1%, and specificity: 86.9%	The proposed pipeline accurately detect and classify focal liver lesions from CEUS images
[132]	99 US images (Cyst: 29; HEM: 37; Malignancies: 33)	Manual ROI selection by an experienced radiologist	FOS, GLCM, Law’s TEM, and echogenicity. PCA for dimensionality reduction	ANN	Accuracy (Cyst vs. HEM): 99.7%, Cyst vs. Malignant: 98.72%, and HEM vs. Malignant: 96.13%	ANN showed superior performance compared to other ML algorithms such as SVM in diagnosing focal liver lesions
[133]	88 patients providing 111 US images (95 FLLs and 16 normal)	Manual ROI selection and image enhancement	Textural features	SVM	Overall one-against-one gives accuracy of 93.1%	One-against-one approach using multi-class SVM provides better results compared to tree structured approach
[134]	110 US images (Cyst: 44; HEM: 18; HCC: 32; Normal: 16)	ROI selection using level set method and Fuzzy c-means clustering	Stacked Sparse Auto-encoders (SSAE)	NB, KNN, Multi-SVM, Softmax classifier	Overall accuracy: 97.20%, Sensitivity: 98%, Specificity: 95.70%	SSAE features can capture high-level feature representations for diagnosis of focal liver lesions
[135]	364 US images (Abscess: 48; Cirrhosis: 40; Cyst: 30; Echinococcosis: 40; Fatty: 34; HEML 34; Hepatitis: 27; Hepatomegaly: 38; MET: 42)	Active contour segmentation (semi-automatic method) for ROI selection	Textural features in spatial and frequency domain	RF	Overall accuracy for 10-class classification: 91%	Wavelet filtering on US images helps to overcome brightness and contrast variations, in turn helps to improve overall classification performance
[136]	140 US images (Normal: 78; Benign: 26; Malignant: 36)	CHAHE for noise removal and contrast enhancement	Bi-dimensional empirical mode decomposition (BEMD) based features, ANOVA for feature selection	SVM, LDA, KNN, RF	Accuracy: 92.95%, Sensitivity: 90.80%, and Specificity: 97.44%	Proposed model can accurately diagnose FLLs without manually selecting region of interest in liver images
[137]	177 patients with FLLs	Manual ROI selection of lesion areas	Sparse representation features and iterative approach for feature selection	SVM	AUC of 0.94 for classifying benign vs malignant FLLs	Multi-modal US images improves FLL diagnosis
[138]	93 patients providing 47 FLL cases and 46 benign cases	Manual ROI selection across three phases: arterial phase, portal venous phase, and delayed phase	Textural features extracted from each ROI. Deep Canonical Correlation Analysis (DCCA) for feature selection	Multi-kernel learning	Accuracy: 90.41%±5.80	Deep Canonical Correlation Analysis helps to learn better feature representation and explore the correlated information between various views of data, in turn providing superior performance
[139]	4,420 CEUS videos (HCC: 2,110; FNH: 2,310)	Manual ROI selection by an experienced radiologist		3D CNN	Overall accuracy: 93.1%, Sensitivity: 94.5%, Specificity: 93.6%	Extending 2D CNN models to 3D CEUS videos can further improve diagnostic performance of FLLs
[140]	367 US images (Homogeneous: 258; Angioma: 17; MET: 48; HCC: 6; Cyst: 30; HNF: 8)		ResNet-50 and DenseNet-121 models for feature extraction	FCNet	Mean AUC score for 5 lesion types: 0.916	Supervised attention helps the model to focus its attention for prediction as well as interpretability of results
[141]	2,143 patients providing 24,343 US images	Manual ROI selection, image resizing		ResNet-18	AUC for FLLs: 0.924, Sensitivity: 86.5%, Specificity: 85.5%	Proposed model provided superior results to that of skilled radiologists
[142]	15,296 US images (10,687: normal and 4,609: FLL)	ROI selection, image resizing, noise filtering	GWT, LBP and CNN features	SVM	Overall accuracy of 98.40%	Fusing traditional features with CNN features improves FLL diagnosis performance
[143]	20,432 US images containomg HCC, cysts, HEM, and focal fatty sparing	Fan-shaped ROI selection, data augmentation		RetinaNet with ResNet-50 as a backbone	Overall detection rate of 87.0%, sensitivity of 83.9%, and specificity of 97.1%	CNNs has shown good performance in the detection and diagnosis of FLLs in US images
[144]	574 patients providing CEUS images	Image resizing, data augmentation		ResNet-152	Overall AUC of 0.934 and accuracy of 91.0%	DL applied to multi-phase CEUS image overcomes interobserver subjectivity
[145]	91 patients providing CEUS videos for FLLs	ROI selection		CNN	Overall accuracy: 88%	DNN showed superior performance for diagnosing FLLs
[146]	3,873 patients (Cyst: 1,214; HEM: 1,220; MET: 1,001; HCC: 874)	Semi-automatic segmentation to delineate lesion areas		Customised CNN model	Overall accuracy for joint learning: 82.2%, accuracy for classification only system: 79.8%	Joint classification and segmentation system gave better performance compared to segmentation only and classification only systems
[147]	CEUS videos from 145 participants during arterial phase	ROI selection	CNN features	3D-CNN, CNN-LSTM	Accuracy: 98%	Learning the change of enhancement patterns for CEUS videos is an effective approach to classify HCC and FNH
[148]	87 B-mode and CEUS images (13 benign and 74 malignant)	ROI selection	Textural and spatiotemporal features	ML classifiers	Balanced accuracy: 84%	Combination of spatiotemporal and texture features are important to aid accurate FLL classification

*SVM* Support Vector Machine, *RF* Random Forest, *LR* Logistic Regression, *NB* Naïve Bayes, *ANN* Artificial Neural Network, *KNN* K-Nearest Neighbour, *PNN* Probabilistic Neural Network, *DT* Decision Tree, *ELM* Extreme Learning Machine, *CNN* Convolutional Neural Network, *ROI* Region-of-Interest, *US* Ultrasound, *CEUS* Contrast Enhanced Ultrasound, *SWE* Shear Wave Elastography, *HCC* Hepatocellular Carcinoma, *NAFLD* Nonalcoholic Fatty Liver Disease, *HEM* Hemangioma, *MET* Metastasis, *FLL* Focal Liver Lesions

**Table 16:** Summary of studies on HCC diagnosis and prognosis using liver US images

**Study**	**Dataset(s)**	**Data Pre-processing**	**Feature extraction and Selection**	**Learning method(s)**	**Results**	**Main finding(s)**
[149]	A prospective study involving 442 patients with Child A or B cirrhosis		Patient demographics, clinical data, and laboratory values	Multivariate Cox regression model, Random Forest	The regression model has a c-statistic of 0.61 (95% CI 0.56$-$0.67) whereas ML model (RF) has a c-statistic of 0.64 (95% CI 0.60$-$0.69)	Machine Learning algorithms are superior in accurately identifying patients at high-risk of developing HCC
[150]	268 HCC patients from a centre in Romania			CNN	Overall AUC of 0.935, accuracy of 91%, sensitivity of 0.944 and specificity of 0.884	DL outperforms classical ML methods for HCC prediction
[151]	434 Hepatitis B patients from a centre in China			CNN	AUC of 0.900 on the test set	DL based approach can accurately predict 5-year HCC development risk
[152]	CEUS images of 318 patients			CNN	AUC of 0.84	Proposed model based on dynamic CEUS radiomics performed well in predicting early HCC recurrence
[153]	B-mode and CEUS images of 48 patients affected by HCC	Automatic ROI selection		CNN	AUC of 0.982, accuracy of 98.25%, sensitivity of 98.16%, and specificity of 98.24%	The fusion of B-mode and CEUS images at decision level improves the HCC diagnosis performance
[154]	US images of 60 patients with 61 other malignancies and 112 patients with 120 HCC		Clinical features were also given	CNN	Sensitivity of 78.6% and specificity of 82.6%	US combined with clinical features is valuable in differentiating HCC from OM in the setting of cirrhosis
[155]	1,241 CEUS videos (667 HCC, 574 non-HCC)	ROI selection, data augmentation	Time-intensity curve features	ResNet-50	Accuracy: 83%, AUC: 0.89	Integrating features from different perfusion stages is significant for HCC prediction
[156]	200 US images	Manural ROI selection, data augmentation	Textural features	ML classifiers, CNN	Accuracy: 98.9%, AUC: 0.99	Fusion of CNN and ML classifiers lead to improved results

*SVM* Support Vector Machine, *RF* Random Forest, *LR* Logistic Regression, *NB* Naïve Bayes, *ANN* Artificial Neural Network, *KNN* K-Nearest Neighbour, *PNN* Probabilistic Neural Network, *DT* Decision Tree, *ELM* Extreme Learning Machine, *CNN* Convolutional Neural Network, *ROI* Region-of-Interest, *US* Ultrasound, *CEUS* Contrast Enhanced Ultrasound, *SWE* Shear Wave Elastography, *HCC* Hepatocellular Carcinoma, *NAFLD* Nonalcoholic Fatty Liver Disease, *HEM* Hemangioma, *MET* Metastasis, *FLL* Focal Liver Lesions

## Public datasets and online initiatives for the diagnosis of liver diseases

Most of the reviewed studies in this article use private (in-house) datasets. Recently, the research community has felt the need to release benchmark datasets in the public domain so that computational methods can be fairly compared. The ImageNet dataset [60] has been one of the underlying factors in the success of deep learning in computer vision because it enabled targeted progression and objective comparison of methods proposed by the community around the world. With the same motivation, researchers in the field of biomedical image computing have started sharing their curated datasets publicly to advance research and foster fair comparison of methods. We provide an overview of various liver datasets below:

- **B-mode fatty liver ultrasound**: [103] released a B-mode US dataset for the diagnosis of NAFLD steatosis assessment using ultrasound images. It contains 550 B-mode ultrasound scans and the corresponding liver biopsy results. The dataset was collected from 55 subjects admitted for bariatric surgery in the Department of Internal Medicine, Hypertension and Vascular Diseases, Medical University of Warsaw, Poland.
- **SYSU-CEUS**: The SYSU-CEUS dataset [157] contains 353 CEUS videos of three types of focal liver lesions, namely, 186 instances of Hepatocelluar carcinoma (HCC), 109 instances of Hemangioma (HEM), and 58 instances of Focal Nodular Hyperplasia (FNH). Datasets specific to liver tumours have also been made available through participation in online challenges.
- **LiTS**: The Liver Tumor Segmentation Challenge(LiTS) [158] dataset provides 201 contrast-enhanced 3D abdominal CT scans and segmentation labels for liver and tumour regions. Each slice of the volume has a resolution of 512 x 512 pixels. Out of 231 volumes, 131 carry their respective annotations whereas no ground-truth labels are provided for the test set containing 70 volumes. The in-plane resolution ranges from 0.60 mm to 0.98 mm, and the slice spacing from 0.45 mm to 5.0 mm.
- **SLIVER07**: The Segmentation of the Liver 2007 (SLIVER07) [159] dataset is a part of the grand-challenge organised in conjunction with the MICCAI 2007 for liver tumor segmentation. The training data consists of 10 tumors from 4 patients with their ground-truth segmentations. For the test set, consisting of 10 tumors from 6 patients, the ground-truth was not made available to public by the task organisers. The dataset contains liver tumor CT images corresponding to portal phase of a standard four-phase contrast enhanced imaging protocol.
- **3D-IRCADb**: The 3D Image Reconstruction for Comparison of Algorithm Database (3D-IRCADb) 1 consists of 3D CT scans of 10 men and 10 women having liver tumors in 15 of the cases. The anonymised patient image, labelled image corresponding to ROI segmented, and mask images are given in the DICOM format. The in-plane resolution ranges from 0.57 mm to 0.87 mm, and the slice spacing from 1.6 mm to 4.0 mm.
- **CHAOS**: The Combined (CT-MR) Healthy Abdominal Organ Segmentation Challenge (CHAOS) [160] is an IEEE ISBI 2019 challenge dataset focused on segmentation of healthy abdominal organs from CT and/or MRI. The CHAOS dataset contains abdominal CT of 40 subjects having healthy liver. Each slice has a resolution of 512 x 512 pixels.
- **Multi-organ abdominal CT reference standard segmentation**: The Multi-organ Abdominal CT Reference Standard Segmentation dataset [161] comprises 90 abdominal CT images delineating multiple organs such as spleen, left-kidney, gallbladder, esophagus, liver, stomach, pancreas, and duodenum. The abdominal CT images and some of the reference segmentations are from two datasets: The Cancer Image Archieve (TCIA) Pancreas-CT dataset [162] and the Beyond the Cranial Vault (BTCV) abdominal dataset [163]. The segmentation of various organs across these CT volumes was performed by two experienced undergraduate students and verified by a radiologist on a volumetric basis.
- **DeepLesion**: The DeepLesion [164] dataset, released by the National Institute of Health (NIH) consists of more than 32,000 annotated lesions identified on CT images, collected from 4,400 unique patients. Each of the 2D CT scans is annotated with lesion type, bounding box, and metadata. Each images has a resolution of 512 x 512 pixels.
- **MIDAS**: The MIDAS liver tumor dataset from the National Library of Medicine (NLM)’s Imaging Methods Assessment and Reporting project provides 4 liver tumors from 4 patients with five expert hand segmentations. The dataset was made available by Dr. Kevin Cleary at the Imaging Science and Information Systems, Georgetown University Medical Center.
- **CLUST**: The Challenge on Liver Ultrasound Track (CLUST) [165] provides a dataset for automatic tracking of liver in ultrasound volumes. The dataset consists of 86 independent studies, with 64 (2D + t) and 22 (3D + t) studies. The dataset was split into training (40% of all sequences) and testing set (60%) from the complete dataset. Annotations were provided for the training set but no ground-truth provided for the test set.

## Limitations and future directions

In this section, we outline limitations of existing studies based on our extensive literature review. We also propose future research directions to overcome these limitations.

### Limitations

- **Focus on classification** Most of the studies focused on the classification task, i.e., binary classification such as: *Normal* vs. *Fatty*, *Normal* vs. *Fibrosis*, *Normal* vs. *Cirrhosis* or multi-class classification such as: *Normal* vs. *Fibrosis* vs. *Cirrhosis*, *Normal* vs. *Hepatocellular Carcinoma* vs. *Metastasis* vs *Hemangiomas*. However, very little work has been done on disease progression and severity scoring for diseases.
- **Small in-house datasets** Although there has been a lot of work on diagnosing liver diseases using various imaging modalities such as ultrasound, CT, and MRI, there are only a few publicly available datasets. Most studies in our literature review worked on in-house data which are often of small size. Also, these datasets suffer from a class-imbalance problem. As accuracy score is not a reliable metric on imbalanced datasets, the lack of publicly available benchmark datasets often limits the true assessment of proposed algorithms in these studies.
- **Classical CAD system still prevalent** Currently, deep learning has shown tremendous performance improvement across various fields such as computer vision, natural language processing, robotics, and biomedical image processing. Specifically in biomedical image computing, deep learning has shown superior results on various tasks such as *classification*, *segmentation*, and *tracking*. Due to the lack of publicly available large-scale annotated datasets on liver US studies, the classical machine learning pipeline is still prominent in the community.

### Future research direction

- **Need for multidisciplinary approach**: The management of HCC encompasses multiple disciplines including hepatologists, diagnostic radiologists, pathologists, transplant surgeons, surgical oncologists, interventional radiologists, nurses, and palliative care professionals [166]. A study by [167] showed that the development of true multidisciplinary clinic with a dedicated tumour board review for HCC patients increased survival; due to improved staging and diagnostic accuracy, efficient treatment times and increased adherence to clinical diagnostic and therapeutic guidelines. Therefore, the AASLD recommends referring HCC patients to a centre with multidisciplinary clinic.
- **Make use of multi-modal data**: Current state-of-the-art deep learning models when trained on multi-modal data such as B-mode images, Doppler images, contrast-enhanced ultrasound images, and SWE images could improve the early staging and diagnosis of HCC. Multi-modal data can provide complementary information, in turn helping models to improve.
- **Need for benchmark datasets**: In order to push the community’s effort in improving diagnostic performance by proposing novel methods, there is a need to establish a benchmark environment with the release of a large-scale annotated dataset in the public domain. Similar to benchmark algorithms during challenges, the task organisers can release annotated training and validation data but not release test data labels. Once participants have fine-tuned their methods, they may submit predictions on the test set on the challenge evaluation server.

## Conclusion

HCC-related morbidity and mortality continues to up-trend due to delays in diagnosis and treatment as early disease is often asymptomatic. Ultrasound is the recommended first-line imaging modality for diagnosis of chronic liver disease and to screen for HCC; however, contrast-enhanced studies are required to confirm HCC diagnosis. In this paper, we first provide an overview of current diagnostic methods for stages of liver disease. We then lay the foundation of methods such as Image pre-processing, feature extraction, and classification for classical machine learning algorithms and a brief overview of convolutional neural networks, which are specialised deep learning algorithms for processing 2D or 3D data. Then, we reviewed the use of these methods as diagnostic tools in chronic liver disease and HCC. We also discussed the studies reviewed in the survey. Finally, we provide future research directions in assisting diagnostic accuracy and efficiency in clinical workflow. We believe that by adapting AI technologies into medical radiology, diagnostic imaging tools have the potential to be implemented in first-line management of chronic liver disease and HCC.

## Footnotes

1 https://www.ircad.fr/research/3dircadb/
